# Climatic and geological drivers of diversity in Iranian Barbels lineage (Cypriniformes: Cyprinidae: Barbinae and Torinae): An integrative taxonomic perspective

**DOI:** 10.1371/journal.pone.0349868

**Published:** 2026-06-11

**Authors:** Hadi Khoshnamvand, Asghar Abdoli, Faraham Ahmadzadeh, Karel Janko

**Affiliations:** 1 Department of Biodiversity and Ecosystem Management, Environmental Sciences Research Institute, Shahid Beheshti University, G.C., Evin, Tehran, Iran; 2 Laboratory of Non-Mendelian Evolution, Institute of Animal Physiology and Genetics of the CAS, Libechov, Czech Republic; 3 Department of Biology and Ecology, Faculty of Science, University of Ostrava, Ostrava, Czech Republic; Universität für Bodenkultur Wien: Universitat fur Bodenkultur Wien, AUSTRIA

## Abstract

Iranian Barbel taxonomy and evolutionary history (Cyprinidae: Barbinae and Torinae) remain contentious due to overlapping morphological traits and limited molecular data. This study applies an integrative taxonomic framework to elucidate species boundaries, phylogenetic relationships, and the environmental drivers of diversification within Barbels lineages across Iran. We analyzed mitochondrial DNA (*Cytb* and *COI* genes), seven meristic morphological characters, and five spatial environmental predictors from specimens collected across localities representing major Iranian basins. Phylogenetic reconstructions using Maximum Likelihood and Bayesian Inference revealed three main monophyletic groups: (1) *Arabibarbus*, *Mesopotamichthys*, and *Carasobarbus* (Torinae); (2) *Luciobarbus*; and (3) *Barbus* sensu stricto (Barbinae). Principal Component and Canonical Variate Analyses of meristic data corroborated molecular findings, supporting the delineation of this taxa. Ecological Niche Evolution analysis indicated several species occupy similar climatic niches, suggesting parallel evolutionary responses to environmental pressures. Divergence time estimates and lineage-through-time analyses linked major cladogenic events to regional orogeny and Quaternary climatic fluctuations. Species delimitation analyses suggested potential synonymy among specific taxa (e.g., *L. capito* with *L. conocephalus*; *L. esosinus* with *L. xanthopetrus*), highlighting the need for taxonomic revision. Our integrative approach demonstrates that geological history and climatic factors have shaped the diversity and distribution of Barbels in Iran. These findings provide a robust framework for future taxonomic, conservation, and biogeographic studies of Iranian freshwater fishes.

## Introduction

Because species are key and central to ecology, evolution, conservation, and biogeography research, experts express that they are the fundamental units in biology [1]. For this reason, the definition and identification of species have been debated since the beginning of systematic biology [2–4]. Modern systematics examines Earth’s biodiversity and phylogenetic connections, primarily aiming to discover and describe new species [5,6]. Traditionally, species identification relied on distinguishing morphological distinctions, whether typological or quantitative. These distinctions continue to be regarded as essential evidence by numerous biologists [7–9].

In certain situations, relying solely on morphological characteristics to diagnose species can be challenging or unattainable [10–12]. In recent years, development in DNA sequencing has dramatically enhanced our ability to detect cryptic species, resulting in a significant increase in detection rates [13–15]. Additionally, through phylogeographic analyses, we could have uncovered substantial levels of phylogenetic diversity [16,17]. Similarly, European Barbels have recently been investigated using mtDNA [18,19].

A recent checklist was published by Sayyadzadeh and Esmaeili [20] and Eagderi et al. [21] show that Iran’s freshwater fishes exhibit remarkable diversity. Within Iran’s inland water bodies are 300 species belonging to 110 genera, 36 families, 23 orders, and three classes [20]. These species are spread across 19 main basins, showcasing the rich aquatic biodiversity of Iran. In terms of both abundance and species diversity, cyprinid fishes (Cyprinidae) play a significant role in the Eurasian temperate freshwater fish fauna [22–25] as they are the dominant group, comprising approximately more than 3000 species worldwide [26,27]. Based on a recent checklist, the Cyprinidae family, with 74 confirmed species in Iran, has the highest species richness in a single family [20]. Barbels group belonged to Cyprinidae, and in recent years, researchers have always debated the identification and validity of its species. In 1841, Heckel was the first to describe around 12 species of Barbus in freshwater in Iran. Despite the existence of numerous publications on the taxonomy status of Barbus, the available data set for Barbus fish assemblages remains limited. Since 1998, when Coad classified all the Iranian fish species of Barbus under a single genus called Barbus, this group has experienced many changes in its taxonomic status and the number of identified individuals, as there has yet to be a complete agreement on the taxonomic status of the Barbels group. For example, some experts put all the Iran populations in one genus and others put the group in several genera, subgenera and subfamilies [28–40].

Thus far, 18 species from the Barbels taxa in two subfamilies (Barbinae and Torinae) and five genera, including *Carasobarbus* Karaman, 1971 [41], *Arabibarbus* Borkenhagen, 2014 [28], *Luciobarbus* Heckel, 1843, *Mesopotamichthys* Karaman, 1971 and *Barbus* Cuvier, 1816 sensu stricto (str), have been reported from different basins in Iran region. Nevertheless, there is still a disagreement about the validity of the group in Iran, and experts have not reached an agreement about the species status of the group (see; Coad, [31]; Eagderi et al., [34]; Esmaeili et al., [35]; Jouladeh-Roudbar, [36]; Sayyadzadeh & Esmaeili [20]; Valiallahi, [42]).

Despite ongoing taxonomic revisions, the species boundaries and phylogenetic relationships within Iranian Barbels (Barbinae and Torinae) remain unresolved due to limited integrative studies that combine molecular, morphological, and ecological data. Furthermore, the role of climatic and geological factors in shaping the diversification and current distribution of these lineages has not been comprehensively investigated across Iran’s diverse freshwater basins; we were driven to investigate this group using an integrated approach. Therefore, in the current study, we investigate i) whether the recognized species currently exhibit genetically distinct lineages, ii) the phylogenetic relationships among the putative species, and iii) the geological and climatological processes associated with shaping diversity and distribution, and how climatic niche evolution occurs during speciation.

## Materials and methods

### Ethical compliance

This study involved live vertebrate animals (freshwater fishes) and was performed in strict compliance with Iranian national regulations for animal research (Iranian Animal Welfare Act, 2005) and the Guidelines for the Use of Fishes in Research published by the American Fisheries Society (2014). The research protocol was approved by scientific collection permit No. 400/213/41 from the Iranian Department of Environment, which also covers euthanasia procedures.

### Taxon sampling and laboratory procedures

**Fig 1:**
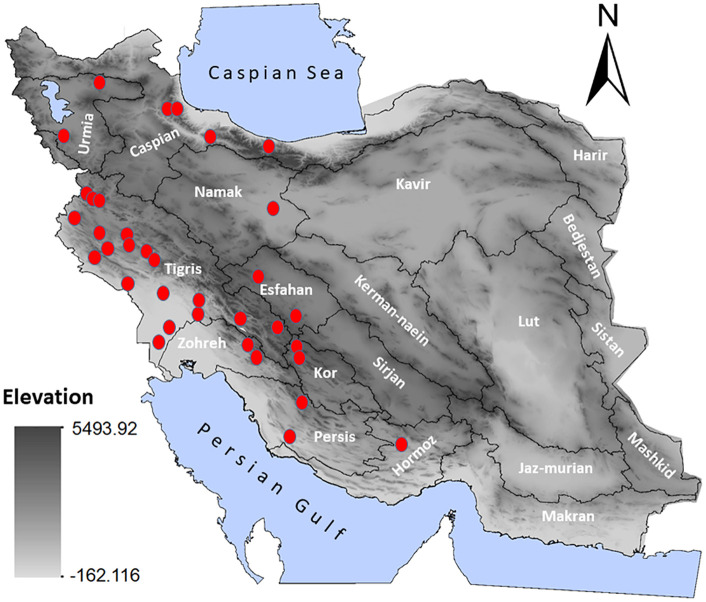
The distribution of Iranian Barbels across 19 basins. The red circles represent the locations of samples used for genetic and meristic analysis.

Fish specimens were collected from 36 different localities throughout its distribution range in Iran to gather comprehensive phylogeny data for the Barbels group. Multiple methods, such as electric fishing gear, cast nets, and hook and line, were employed for collection. The sampled basins included Caspian, Urmia, Tigris, Namak, Esfahan, Zohreh, Persis, Kor, and Hormoz (Fig 1), representing a wide geographic representation. After identification following Abdoli [43], fish were euthanized prior to tissue sampling. Euthanasia was performed by immersion in an overdose of tricaine methanesulfonate (MS-222; Sigma-Aldrich, USA) at a concentration of 300 mg/L buffered with an equal amount of sodium bicarbonate (NaHCO_3_) to maintain pH at 7.0–7.5. Fish were left in the solution for a minimum of 10 minutes after cessation of opercular movement to ensure death. Confirmation of euthanasia was based on the absence of gill movement, lack of response to gentle tactile stimulation of the caudal peduncle, and loss of vestibulo-ocular reflex. Following confirmation, a clip of the left pectoral fin (approximately 2–5 mm²) was removed for genetic analysis. Clips were then preserved in 90% ethanol to maintain the integrity of the genetic material. Specimens were held in the Biodiversity and Ecosystem Management molecular ecology lab at Shahid Beheshti University, Iran. Collection and locality data for sampled fishes are described in S1 Table.

### DNA extraction, amplification and sequencing

For DNA extraction, the high-salt method as described by [44] was utilized. The cytochrome b gene (*Cytb*) was chosen for molecular analysis. To amplify the targeted gene, the forward primer F08_F (5′ GACTTGAAAAACCACCGTTG-3′) and the reverse primer E08_R (5′ CTCCGATCTCCGGATTACAAGAC −3′) were employed, as suggested by Wang et al. in 2021. The amplified fragment length is 513 base pairs (bp). Based on alignment with the complete mitochondrial genome of *Barbus barbus* (GenBank accession NC_025332.1), the amplified region corresponds to nucleotide positions 14,125–14,637 of the *Cytb*. The Polymerase Chain Reactions (PCRs) were carried out using 1 μl of template DNA (50–100 ng), 0.5 μl of each primer, 12.5 μl of Master Mix Red (Ampliqon), and 10.5 μl of ddH2O to make a total of 25 μl of reaction mixture. The PCR amplification, performed on an MJ Mini™ thermocycler (Bio-Rad), began with an initial denaturation at 95°C for 2 minutes. This was followed by 35 cycles, each consisting of a second denaturation step at 95°C for 1 minute, annealing at 56°C for 30 seconds, and elongation at 72°C for 30 seconds. The final step was a concluding elongation at 72°C for 10 minutes. The PCR product quality was assessed using a 1% agarose gel stained with Safe-Red™. The appropriate amplicons were then sent to Pishgam Inc. for purification and sequencing.

In the current study, alongside the newly generated sequences, additional sequences were obtained from GenBank to enhance taxonomic coverage and phylogenetic resolution. A comprehensive search of GenBank (accessed January 2024) was performed using the keywords “Barbus,” “*Luciobarbus*,” “*Carasobarbus*,” “*Arabibarbus*,” “*Mesopotamichthys*,” “*Cyprinidae*,” combined with “*Cytb*” and “*COI*.” The initial search returned 387 sequences (214 *Cytb*, 173 *COI*) from the target genera and closely related outgroups. Following quality filtering (see Alignments and Phylogenetic Analyses section), 61 sequences were retained for downstream analyses. S1 Table, S9 Data and S10 Data, provides full details of all sequences used, including newly generated and GenBank-derived sequences. Of the 387 initial GenBank hits, 189 were excluded due to short length (<400 bp; n = 94), ambiguous bases (n = 43), lack of geographic data (n = 28), or suspected misidentification based on preliminary phylogenetic placement (n = 24). An additional 137 sequences were excluded after redundancy reduction (identical or near-identical haplotypes). The final dataset comprised 61 GenBank sequences (31 *Cytb*, 30 *COI*) for sequences in the concatenated alignment.

### Alignments and phylogenetic analyses

New sequences were edited using Geneious Prime® V. 2023.1.0 (Biomatters, www.geneious.com). We merged our recently generated sequence data with previously published sequences to establish the evolutionary relationships among Barbus (sl) lineages across its range. *Garra rufa* and *Capoeta capoeta* was considered as outgroup. MAFFT v.6 [45] (https://mafft.cbrc.jp/; algorithm: Auto; scoring matrix: 200Pam/k = 2; Gap open penalty: 1.53) was used to align the datasets of all genes, which were subsequently merged to create a final alignment of 1122 bp (*Cytb*: 513 bp, *COI*: 609 bp). In addition, MrModeltest v.2.3 (Nylander, 2004) with AIC criterion [46] was used to select each gene’s most suitable nucleotide substitution models resulting in the best fit of *Cytb* by the HKY + I + G (I = 0.4368, G = 1.9974) and COI by the GTR + I + G (I = 0.5142, G = 1.5868). IQTree v.1.6.12 [47] was utilized to conduct Maximum Likelihood (ML) inference. The ML analysis was carried out under the GTR + I + G evolutionary model. To assess the confidence of branch supports, the ultrafast Bootstrap (UFB) approach [48] was employed using 1000 pseudoreplicates. The combined dataset was subjected to Bayesian Inference (BI) analysis using MrBayes v.3.2 [49]. The partitioning scheme employed for the Maximum Likelihood (ML) analysis was also used for the BI analysis. The analysis was conducted in two separate trials using five chains for a total of six million generations. Trees and parameters were saved every 100 iterations, resulting in a total of 60001 trees throughout the analysis. In the end, a burn-in phase was implemented where 10% of the trees were discarded. The remaining trees were then utilized to construct the consensus tree using the majority-rule approach, with a threshold set at 50%. The split frequencies exhibited a final standard deviation (SD) of 0.0015, with parameters calculated individually for each partition. To assess convergence and evaluate the performance of each run, Tracer v.1.6 [50] was used. To assess the statistical significance of different tree topologies, the Shimodaira-Hasegawa (SH) test was utilized. This was done through a likelihood ratio test with 1000 bootstrap pseudoreplicates (SH-aLRT), as implemented in IQ-Tree v.1.6.12 [51,52]. In order to analyze the genetic distances among clades, uncorrected calculations were performed using Mega X [53] on separate mtDNA datasets for *Cytb* and *COI*.

### Network analysis

We conducted a haplotype network analysis using two mitochondrial datasets to determine and visually represent the phylogenetic relationships within the Barbels taxa. We utilized NETWORK v.10.2 [54] to construct a median-joining (MJ) network, allowing us to identify the potential origins of each detected specimen.

### Species delimitation

The General Mixed Yule Coalescent (GMYC) model [55] and Bayesian implementation of the Poisson tree processes model (bPTP: [56] were employed to define the delimiting of the Barbel species. This was done by analyzing the mtDNA sequence dataset consisting of *Cytb* and *COI* genes. The GMYC model was executed using the R package *SPLITS*, which stands for SPecies’ Limits by Threshold Statistics. This method can be accessed through the R package ‘splits,’ which can be found at the following link: https://r-forge.rproject.org/projects/splits/.

### Estimation of divergence times

Divergence times were estimated using the combined dataset (two genes, 1122 bp) with BEAST v.1.7.2 [57]. Divergence times were estimated using a secondary calibration approach based on the estimated age of the Barbels taxa (approximately 30 Mya; [58,59]. A lognormal prior distribution was applied to the Barbels crown node, with a mean of 30 Mya and a 95% highest posterior density (HPD) interval spanning 25–35 Mya (offset = 20, mean in real space = 10, standard deviation = 0.5). The lognormal distribution was chosen because it allows for a small probability of older ages while preventing unrealistically deep divergences, consistent with standard practice in molecular clock analyses [60]. This prior reflects uncertainty in the secondary calibration while remaining biologically plausible given the fossil record of cyprinids.

Additionally, the Yule model was used as the speciation prior. The analysis was run for 60 million generations and sampled every 1,000 generations. Tracer version 1.6.1 was used to assess the MCMC analyses’ convergence diagnostics. Lineage through Time plotting (LTT) was created in Barbels group using Tracer version 1.6 to show the diversification of extant lineages over time. The LTT plot was constructed based on the combined dataset comprising two genes.

To assess the robustness of our divergence time estimates to the choice of calibration, we performed a sensitivity analysis using three alternative approaches: (i) excluding the Barbels secondary calibration and using only a loose upper bound (90 Mya) based on the oldest cypriniform fossils; (ii) using a different secondary calibration for the Barbels node (28 Mya) from an alternative published study [61]; and (iii) applying a uniform prior (25–35 Mya) instead of a lognormal distribution. Results from these sensitivity runs showed that while absolute ages shifted by ±2–5 Mya, the relative order of divergences and the inference that major cladogenetic events occurred during the Oligocene–Miocene remained consistent. The 95% HPD intervals overlapped substantially across all runs. These cross-validation results indicate that our main conclusions regarding the temporal link between diversification and Neogene geological events are robust to the choice of calibration point, although absolute ages should be interpreted with caution (S4 Table).

### Morphology analysis

For the morphology of the study, we use the meristic characters because, based on Talwar and Jhingran [62] and Nelson, J. S. (2006) [63], countable characteristics are usually more important than measurable characteristics, and they are less affected by the environment and the age of the fish.

A total of 90 specimens were identified as *Arabibarbus grypus*, *Mesopotamichthys sharpyei*, *Carasobarbus sublimus*, *Carasobarbus kosswigi*, *Carasobarbus luteus*, *Luciobarbus capito*, *Luciobarbus kersin*, *Luciobarbus xanthopterus*, *Luciobarbus esocinus*, *Luciobarbus barbulus*, *Luciobarbus mursa*, *Luciobarbus subquincunciatus* (DOE Lurestan Museum), *Barbus lacerta*, *Barbus cyri*, and *Barbus karunensis* were examined for seven meristic characters (File 1. S2 Table). For several species that we missed, meristic characters were obtained from reliable scientific sources such as [43] and Published personal notes by [64]. These species contains: *Luciobarbus brachycephalus*, *Luciobarbus conocephalus*, and *Barbus miliaris*.

The analyzed meristic characters were the following:

Dorsal fin unbranched rays (Dfur), Dorsal fin branched rays (Dfbr), Anal fin unbranched rays (Afur), Anal fin branched rays (Afbr), Pectoral fin branched rays (Pfbr), Lateral line scales (Lls), and Pharyngeal teeth (Pt). All specimens are preserved in the Biodiversity lab, Environmental Sciences Research Institute, Shahid Beheshti University, Tehran, Iran. Prior to PCA, meristic data were standardized using z-score transformation (mean = 0, SD = 1) to account for differences in character ranges. This scaling ensures that variables with larger numerical values (e.g., lateral line scales) do not disproportionately influence the ordination. PCA was performed using the ‘*FactoMineR*’ package in R. Eigenvalues and proportion of variance explained are reported for each principal component.

Additionally, Canonical Variate Analysis (CVA) using the ‘*MASS*’ package was employed to confirm the expected morphological divergence and validate the generic assignments between the genera under investigation. Analyses were performed with R v.4.1.3 [65].

### Ecological Niche evolution

To quantify climatic niche differentiation among Barbus group species, we combined ecological niche modeling (ENM) with formal niche overlap tests. Occurrence data for each species (minimum 5 unique localities) were obtained from field sampling (S1 Table) and complemented with records from GBIF and published literature. Five bioclimatic variables (BIO1 = Annual Mean Temperature, BIO6 = Min Temperature of Coldest Month, BIO7 = Temperature Annual Range, BIO12 = Annual Precipitation, BIO14 = Precipitation of Driest Month) at 2.5 arc-second resolution were extracted from WorldClim v.2.1. To reduce multicollinearity, variables with Pearson correlation |r| > 0.8 were excluded, retaining BIO1, BIO7, and BIO14 for final models. For each species with ≥5 occurrences, we built MaxEnt models (v.3.4.4) using 75% of localities for training and 25% for testing (10 bootstrap replicates). Model performance was evaluated using the Area Under the Receiver Operating Characteristic Curve (AUC) and the True Skill Statistic (TSS). AUC values ≥0.7 were considered acceptable, ≥ 0.8 good, and ≥0.9 excellent. TSS was calculated as (sensitivity + specificity – 1), with values >0.5 indicating useful models. Niche overlap between each species pair was quantified using *Schoener’s D* and *Hellinger’s I* metrics (range 0 = no overlap to 1 = identical) implemented in the *ecospat* R package. To test whether observed overlaps differ from random expectations, we performed: Niche equivalency test (identity test): Occurrences of two species are pooled and randomly split into two pseudo-replicates (100 randomizations). Rejection of the null hypothesis (p < 0.05) indicates that niches are not identical.

Niche similarity test (background test); one species niche is compared against randomly selected background points from the other species range (100 randomizations). Rejection indicates niches are more (or less) similar than expected by chance. All analyses were conducted in R v.4.1.3 using packages *dismo*, *ecospat*, *raster*, and *ENMeval*. Results are reported in S6 Table and S7 Table.

To create profiles of predicted niche occupancy (PNO), each climatic variable map was transformed into a histogram consisting of 100 bins of equal intervals, establishing a connection between habitat suitability and the climatic variable bins. To ensure a balanced evolutionary signal for macroevolutionary analysis, we restricted ancestral niche reconstructions to one terminal per species by selecting a single representative from duplicate branches within the Barbels taxon BEAST phylogeny. It is important to acknowledge that this may show a limitation which excludes intraspecific climatic variation from the analysis, which could potentially bias inferences regarding niche evolution. All calculations were performed using the PHYLOCLIM package [66] in R 4.1.3.

## Results

### Phylogenetic analyses

**Fig 2:**
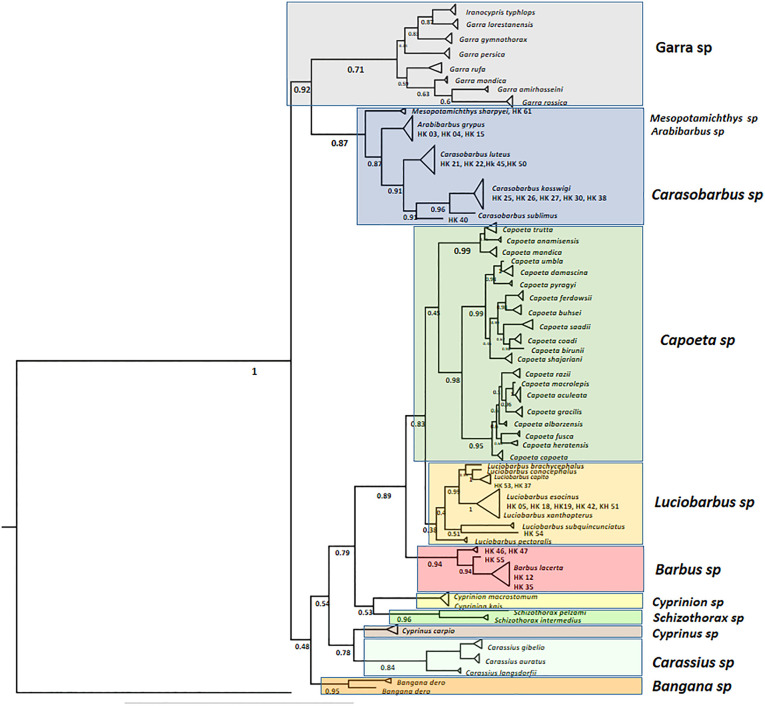
Phylogenetic relationships of Cyprinidae family and current position of Iranian Barbels group based on *Cytb.* Each node indicated BI posterior probabilities.

**Fig 3:**
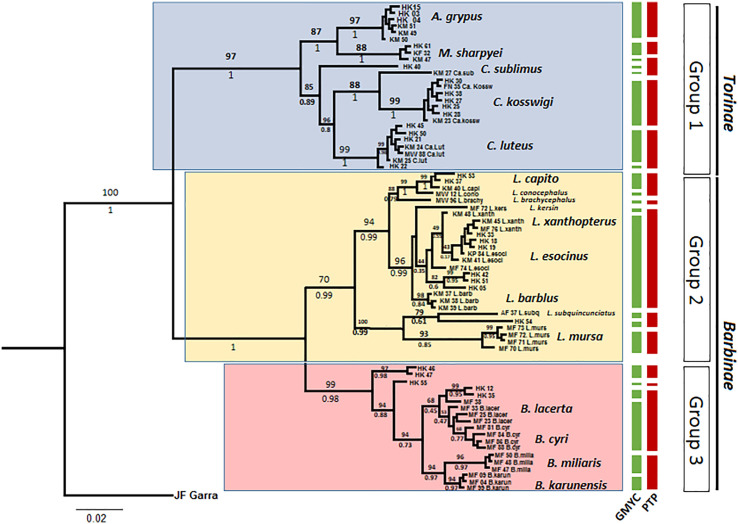
The phylogenetic tree was reconstructed for Iranian Barbels based on the *COI* and *Cytb* genes using MrBayes. For each node, nodal supports indicate BI posterior probabilities (below) and ML bootstrap support (top). Red circles represent samples sequenced in this study.

The study used two methods, maximum likelihood (ML) and Bayesian inference (BI), to create phylogenetic trees from combined genes, and both methods produced the same results. Using the *Cytb* gene, found that the Torinae clade, which contains the species *Carasobarbus*, *Arabibarbus*, and *Mesopotamichthys*, is closely related to the Garra’s clade. Additionally, the *Luciobarbus* and *Barbus* species are closely associated with the *Capoeta* clade (Fig 2). The analysis of the *COI* and *Cytb* genes identified three clades within the Barbels group: Group 1, the Torinae clade, includes several species of *Arabibarbus*, *Mesopotamichthys*, and *Carasobarbus* found in western, southwestern, and southern Iran. Group 2 consists of numerous *Luciobarbus* species from northern, western, and southwestern Iran. Group 3 contains various Barbus species, also from north, western, and southwestern Iran (Fig 3). Essentially, the study confidently classified the Barbels group into three separate, well-supported clades based on genetic analysis.

Detailed information about the clades and genetic distances among species can be found in S3 Table.

The uncorrected genetic distances observed between five reported *Barbus* (sl) genera were around 4–16% for *Cytb* and 4–17% for *COI* (Table 1, and File 4 and File 5).

**Table 1:** Uncorrected genetic p-distances within Barbels clades using COI (below matrix) and *Cytb* (above matrix).

	Carasobarbus	Barbus	Luciobarbus	Arabibarbus	Mesopotamichthys
*Carasobarbus*		0.161	0.131	0.046	0.057
*Barbus*	0.151		0.064	0.172	0.178
*Luciobarbus*	0.153	0.097		0.146	0.142
*Arabibarbus*	0.062	0.160	0.148		0.055
*Mesopotamichthys*	0.067	0.149	0.157	0.041	

For the concatenated mtDNA dataset (1122 bp; n = 151 sequences including outgroups), we identified 387 polymorphic sites (S), of which 312 were parsimony-informative. A total of 89 distinct haplotypes were recovered across all Iranian Barbel lineages (concatenated dataset). Haplotype diversity was high (Hd = 0.96 ± 0.01), while nucleotide diversity was moderate (π = 0.043 ± 0.002) (Table 2).

**Table 2:** Summary statistics for mtDNA datasets (*Cytb*, *COI*, and concatenated).

Dataset	n	Alignment length (bp)	Polymorphic sites (S)	Parsimony-informative sites	Haplotypes (H)	Haplotype diversity (Hd ± SD)
*Cytb* (all)	151	513	179	149	54	0.94 ± 0.01
COI (all)	138	609	208	163	61	0.95 ± 0.01
Concatenated (all)	151	1122	387	312	89	0.96 ± 0.01
Clade 1 (Torinae)	42	1122	98	72	18	0.89 ± 0.03
Clade 2 (*Luciobarbus*)	68	1122	156	124	42	0.98 ± 0.01
Clade 3 (*Barbus* s.str.)	41	1122	87	63	29	0.95 ± 0.02

Note: n = number of sequences; SD = standard deviation; outgroups (*Garra rufa*, *Capoeta capoeta*) included in “all” but excluded from clade-specific calculations.

### Network analysis

**Fig 4:**
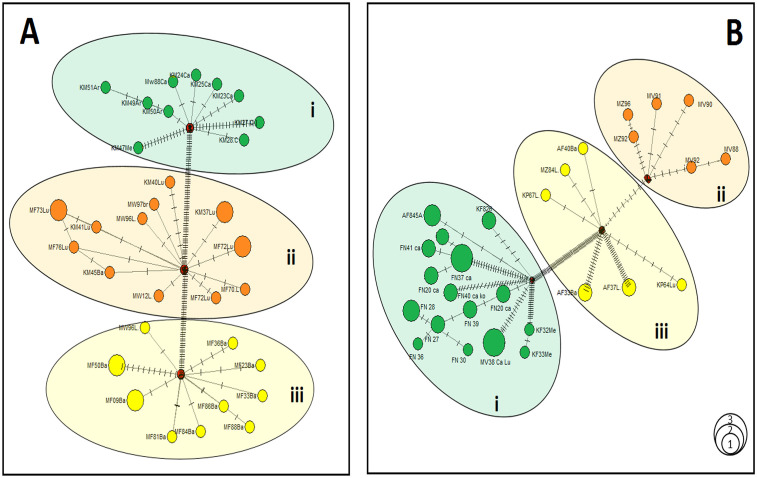
Haplotype networks were constructed using statistical parsimony based on the mitochondrial *Cytb* (a) and *COI* (b) for the Barbels group. Circle size is relative to haplotype frequency.

The haplotype network, created using *COI* and *Cytb* data for *Barbus* (sl) species within their distribution area, revealed the presence of three distinct haplogroups. The network (Fig 4) aligns with the overall structure of the ML and BL trees, confirming its accuracy and reliability.

The examination discovered that each haplogroup is distinctly differentiated from the others by a substantial number of mutations, as visually depicted in Fig 4A and 4B. Furthermore, the Network analysis strongly indicated that nearly all Iranian Barbels taxon can be traced back to the Tigris basin, solidifying their origins in that specific region.

### Species delimitation

**Fig 5:**
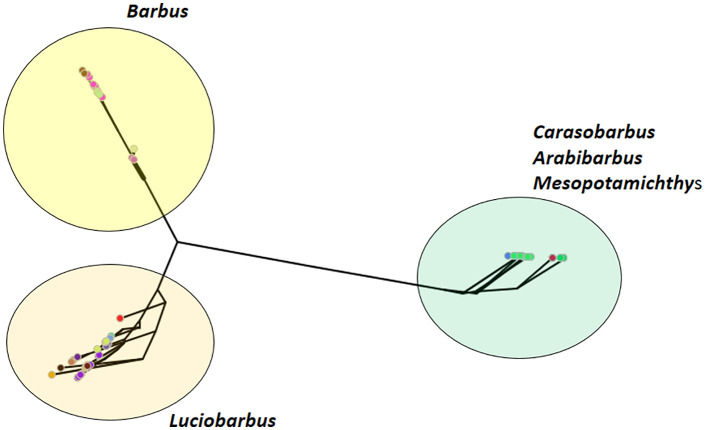
PhyloMap visualization of the PTP (Poisson Tree Processes) model run for species delimitation of the Bl tree based on the concatenated data set for Barbels. 100,000 MCMC generations were used, with the outgroup removed.

Within the Barbels group, the GMYC model detected 22 distinct genetic clusters, whereas the bPTP model applied to the concatenated mtDNA dataset identified 14 clades as distinct (Fig 5). This discrepancy (22 vs. 14) is not unexpected given the known tendencies of these methods: GMYC is prone to over-splitting, particularly when sampling per putative species is limited [67], while bPTP applied to concatenated mitochondrial genes cannot distinguish between incomplete lineage sorting, population structure, and true species boundaries [56,68]. Moreover, reliance on mtDNA alone with its smaller effective population size and maternal inheritance can overestimate divergence compared to nuclear markers [69]. Consequently, we do not interpret either estimate as definitive. Instead, we consider the range of 14–22 as reflecting uncertainty, and we propose taxonomic revisions only for those lineages that receive support from multiple lines of evidence: (i) reciprocal monophyly in both ML and BI phylogenies, (ii) genetic distances >2–3% (following Bagley et al., 2015) [70], and (iii) meristic differentiation where available. The absence of multispecies coalescent approaches (e.g., BPP, STACEY, BEAST) which would require independent nuclear loci is a major limitation of the present study (see Discussion) (Fig 2). In the Phylo-Map plot, the first axis accounted for 67.85% of the variance. In contrast, the second axis accounted for 16.42%. The result depicts that *Carasobarbus*, *Arabibarbus*, and *Mesopotamichthys* genera were grouped on a branch and separated from *Luciobarbus* and *Barbus* (str) genera (Fig 5).

### Estimation of divergence times

**Fig 6:**
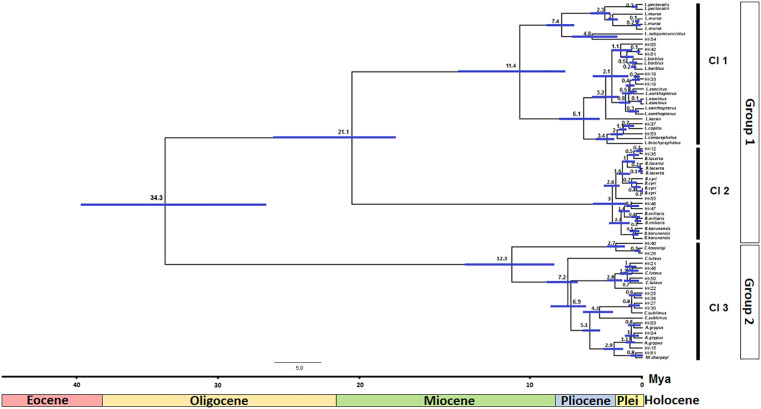
Chronogram with estimated divergence times for the Barbels group based on a secondary calibration approach with the combined COI and *Cytb* gene sequences using BEAST. The nodes’ blue line represents the estimated divergence times, with the 95% highest posterior density (HPD).

According to the calibrated tree, the Barbels group species was divided into two main groups that diverged approximately 34.3 Mya (95% highest posterior density (HPD), ranging from 26.8–40.7 Mya). The diversification within group І happened at 21.1 Mya (95% HPD: 18.6–26.1 Mya); it was divergent in two main clades (Cl 1 & Cl 2). Cl 1 contains *Luciobarbus* species, and Cl 2 contains *Barbus* (str) species. In addition, group ІІ includes *Carasobarbus*, *Arabibarbus,* and *Mesopotamichthys* species (Fig 6).

The LTT plot revealed that diversification within the *Barbus* (sl) species occurred approximately 35 million years ago, showing an increasing slope up to the present (S8 Fig).

**Fig 7:**
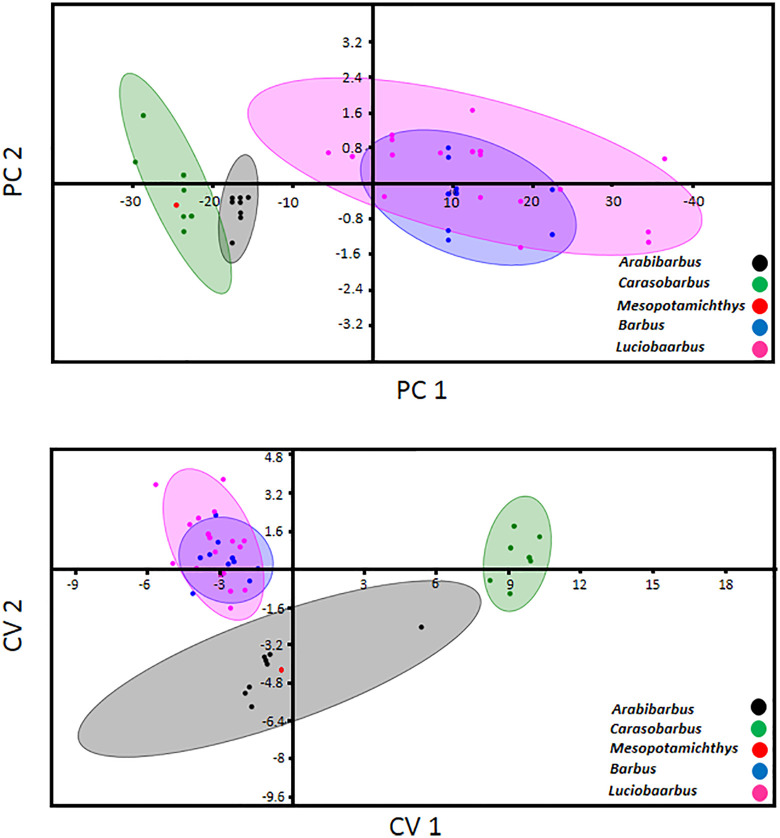
Principal Component Analysis (PCA) and Canonical Variate Analysis (CVA) of meristic characteristics in five genera of the Barbels group. The variables are placed on two axes, PC1 and PC 2, as well as CV1 and CV2.

Prior to PCA, meristic data were standardized (z-score transformation) to account for differences in variable ranges. The first four principal components explained 92.97% of the total variance (PC1: 44.64%, PC2: 26.46%, PC3: 14.11%, PC4: 7.76%; eigenvalues: PC1 = 3.12, PC2 = 1.85, PC3 = 0.99, PC4 = 0.54). Dorsal fin branched rays, pectoral fin branched rays, and lateral line scales showed the highest loadings on PC1 and PC2. The PCA plot (Fig 7) shows that *Luciobarbus* and *Barbus* (str) group together, while *Carasobarbus*, *Arabibarbus*, and *Mesopotamichthys* form a separate cluster (S5 Table). CVA (CV1: 87.23% and CV2: 11.27%) results showed significant differences between the studied genera (P < 0.05) (S2 Table). On the other hand, *Arabibarbus* with *Mesopotamichthys,* and *Luciobarbus* with *Barbus* (str) showed no difference in the CVA (Fig 7). Lateral line scales were the main trait used to separate the genera.

### Ecological Niche evolution

MaxEnt models showed good to excellent predictive performance for all species with sufficient occurrence data (mean AUC = 0.87 ± 0.05, range 0.79–0.94; mean TSS = 0.68 ± 0.09, range 0.55–0.82; full details in S4 Table). BIO14 (Precipitation of Driest Month) was the strongest predictor in 8 of 12 species models, followed by BIO7 (Temperature Annual Range).

Niche overlap statistics: Pairwise niche overlap (Schoener’s D) among the species ranged from 0.11 to 0.89 (mean = 0.52). The highest overlaps were observed between sympatric species in the *Luciobarbus* clade, while the lowest overlaps involved the allopatric species *L. mursa* (vs. *L. esocinus*: D = 0.15) and *B. miliaris* (vs. *C. luteus*: D = 0.11). Niche equivalency tests rejected the null hypothesis of identical niches (p < 0.05) for all except three species pairs (*L. esocinus*–*L. xanthopterus*, *L. capito*–*L. conocephalus*, and *B. lacerta*–*B. cyri*), suggesting that even morphologically similar species occupy statistically distinguishable climatic niches. However, niche similarity tests revealed that for most species pairs (73%), background niche overlap did not differ significantly from random expectations (p > 0.05), indicating that climatic divergence may reflect geographic isolation rather than distinct physiological tolerances (S6 Table and S7 Table).

**Fig 8:**
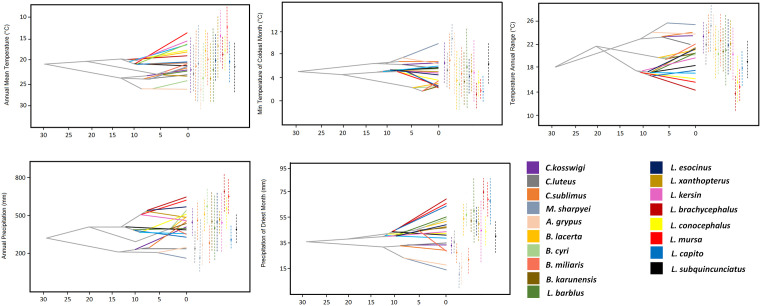
The evolutionary history of climatic tolerances for the Barbels group species group. The chronogram topology of the group is projected into niche parameter space (y-axis), and mean climatic tolerances based on 100 random samples of the PNO profiles are depicted at internal nodes. Each species’ 80% central density of climate tolerance is denoted by a vertical dashed line, with the mean represented by a corresponding point. **(A)** Bio1, **(B)** Bio6, **(C)** Bio7, **(D)** Bio12 and **(E)** Bio14.

The anticipated patterns of niche occupancy profiles displayed significant diversity in specific bioclimatic factors. Taxa within different groups evolved in distinct climate niches, whereas different degrees of overlap among the five variables were observed for most species, as depicted in Fig 7. Several overlapping dashed lines signify similar climatic adaptability among some species across all bioclimatic layers. Among the climatic variables used, it seems that Bio7 and Bio14 had the most critical effect on the separation of Barbels group nich species. The most obvious phylogenetic niche divergence was seen for *L. mursa* and *L. brachycephalus* from lineages of *Luciobarbus*. So, in almost all climatic variables, they showed different climatic niches than other species. As well as, the species distributed across the rivers in the southern basins of Iran exhibit nearly identical climatic niches, particularly within the *Luciobarbus* species clade in the southern regions of the country, such as *L. barbulus*, *L. xanthopterus*, *L. esocinus*, *L. kersin*, *L. subquincunciatus* (Fig 8).

## Discussion

Our study focused on the Iranian Barbels group (Cypriniformes: Cyprinidae: Barbinae and Torinae), aiming to reconstruct its phylogeny and evaluate its species diversity and taxonomy. Acknowledging certain limitations of the mtDNA-based methods employed in this study, the resulting taxonomic system may require further refinement and significant future updates to fully and accurately reflect the true taxonomic realities of these taxa. Nevertheless, our integrative taxonomy analysis serves as a valuable insight and a foundational step to guide future investigations in this field.

### Phylogenetic relationships

Since 2007, researchers have reclassified these species into five different genera, whereas before that year, they all belonged to the same genus. The current phylogenetic trees using mtDNA sequences showed three distinct evolutionary lineages within the Barbels group, as depicted in Fig 2. Furthermore, no overlapping haplotypes were observed for mitochondrial genes, as indicated in Fig 3. All clades were divided into three main groups. The first one comprised *Arabibarbus* + *Mesopotamichthys* + *Carasobarbus* lineages (*A. grypus, M. sharpyei, C. sublimus, C. kosswigi and C. luteus*), the second included *Luciobarbus* clades (*L. capito*, *L. conocephalus*, *L. brachycephalus*, *L. kersin*, *L.xanthopterus*, *L. esocinus*, *L. barbulus*, *L. subquincunciatus*, and *L. mursa*) and third contains *Barbus* (str) lineages (*B. lacerta*, *B. cyri*, *B. miliaris and B. karunensis*).

In the study, among the five reported genera, *Arabibarbus*, *Mesopotamichthys*, and *Carasobarbus* belong to a clade; the genetic distance showed that these three genera have a low genetic distance compared to other genera. Specifically, the lowest genetic distance for the *COI* was among the *Arabibarbus* and *Mesopotamichthys* genera at 4.1%, and *Cytb* was estimated between the *Arabibarbus* and *Carasobarbus* genera at 4.6%. Roul et al. [71] for *Pampus* genera mentioned the genetic distance for divergence value between the two major clades to be more than 8%. In this direction, Galván-Quesada et al. [72] noted that based on *Cytb* for divergence between two clades of *Dormitator* genera, a genetic distance of more than 8.5% is acceptable. Nevertheless, the genetic distance among *Arabibarbus*, *Mesopotamichthys,* and *Carasobarbus* genera for two genes was lower than 8% (Table 1). This shows the affinity of the mentioned genera with each other and having a common ancestor.

Consistent with our phylogeny tree results, [73] reported *Arabibarbus*, *Mesopotamichthys,* and *Carasobarbus* genera in a clade. Furthermore, similar to our phylogeny tree [61,74,75], using *Cytb*, showed that *Arabibarbus* and *Mesopotamichthys* are in the same cluster or group. On the other hand, the position of these three genera in the phylogeny tree of the Cyprinidae family showed that they might not belong to the Barbels taxa. These three genera were placed together with the *Garra* group as a cluster and a monophyletic group, indicating a low affinity with the other Barbels group (Fig 3). To confirm this finding, Coad [64] mentioned that *Arabibarbus* and *Mesopotamichthys* do not belong to Barbels. In addition, *Luciobarbus* and *Barbus s.str.*position in the Cyprinidae family’s phylogeny tree illustrates that they have more affinity with the *Capoeta* group (Fig 2). The phylogeny tree obtained from the study of Levin et al. [58] and Yang et al. [76] was similar to our Phylogenetic tree. The haplotype network analyses of the Barbels group revealed that the haplogroup comprising *Carasobarbus*, *Arabibarbus,* and *Mesopotamichthys* haplotypes exhibited numerous mutations compared to other haplogroups. This observation suggests a distinct separation of the haplotypes within this haplogroup from other haplogroups (Fig 4). The PhyloMap (Fig 5) results in the present study aligned with the results.

Despite identification keys suggesting that certain specimens should cluster together, our genetic and phylogenetic analyses revealed significant discrepancies, indicating potential misidentification or the presence of cryptic species. The KH40 specimen, which was expected to group with *C. sublimus*, instead formed a distinct lineage with a K2P genetic distance of 7% (S11 Data and S12 Data). The GMYC model also separated KH40 from *C. sublimus*, while the bPTP model grouped them together, highlighting a conflict between the species delimitation models. Furthermore, a group of samples (HK05, HK42, and HK51), identified morphologically as *L. barbulus*, formed a separate lineage near the *L. esocinus* clade. This lineage showed a genetic distance of 2.5% from *L. barbulus* and was positioned separately by the bPTP model. Similar patterns of divergence were observed in other samples: Samples HK46 and HK47, identified as *B. lacerta*, were placed in a separate lineage with a 4.6% genetic distance from other *B. lacerta* specimens. The HK55 sample, identified as *B. cyri*, also formed a separate lineage with a 2.8% genetic distance. Finally, the HK35 sample from the Bashar River, previously identified as *B. karunensis* by Khaefi et al. (2017), was placed within the *B. lacerta* clade with a genetic distance of 1.4%. The GMYC model, however, positioned it separately from the *B. lacerta* clade.

Our study found that several species pairs, including *L. capito* and *L. conocephalus*, *L. esocinus* and *L. xanthopterus*, and *B. lacerta* and *B. cyri*, exhibited low genetic distances of less than 2.5% K2P. These species also showed similar phylogenetic relationships, as depicted in Fig 2 and S3 Table. This finding contrasts with previous research using protein, mitochondrial DNA, and morphological characteristics that identified these pairs as distinct clades [36–38,40,42,77,78]. Some of these studies (i.e., Khaefi et al., [37,38,78]; Jouladeh-Roudbar et al., [36]) have even proposed new species based on genetic distances of less than 2%. We emphasize that while genetic distances above ≈2.5% combined with phylogenetic distinctiveness (as seen in specimens like KH40) are used to flag potential cryptic diversity, the consistently low distances (under 2.5%) between these recognized species pairs (e.g., *L. capito* and *L. conocephalus*) challenge the utility of relying solely on single, low genetic distance thresholds for species differentiation in this group.

Bagley et al. (2015) [70] believed that the *Poecilia sphenops* Species Complex putative species are distinct from one another by ≥2% and more frequently ≥3% mean pairwise mtDNA genetic distances. Moreover, [79] mentioned that for the genus *Ilyodon Eigenmann*, the genetic distances of *Cytb* with 2% and lower are challenging to recognize and distinct species.

According to previous reports, two species of *Barbus* s.str were recorded and identified from Iran, i.e., *L. conocephalus* and *B. karunensis* [78,80]. However, our investigation revealed that these species were not grouped separately but rather closely related to the *L.capito* and *B. lacerta* clades, respectively (see Fig 2 and File 1, S3 Table). Besides, [81,82] studied the phylogenetic status of *L. esocinus* and *L. xanthopterus*. They indicated that *L. esocinus* and *L. xanthopterus* are phylogenetically closely related. They mentioned no significant genetic distance between these two species (under 0.7% K2P distance). This was in line with the results of the present study (Fig 3 and S2 Table).

The current study’s findings revealed that within the *Barbus* (sl), 22 lineages were identified by GMYC, and bPTP identified 14. According to [67], the outcomes of the GMYC model might potentially overestimate the number of species. Additionally, it could create clusters identified as distinct species even when the sampling of demes is less than approximately 20%. However, the bPTP model is considered more cautious in its estimations.

The discrepancy between GMYC (22 clusters) and bPTP (14 clusters) highlights a well-known challenge in DNA-based species delimitation: different algorithms can yield substantially different results, especially when applied to single-locus mitochondrial data [83,84]. While GMYC tends to over-split when sampling is incomplete [67], bPTP is not immune to errors; both methods are sensitive to the underlying phylogeny and cannot account for gene tree-species tree discordance due to incomplete lineage sorting or introgression [56,68]. A more significant limitation of our study and one that we must emphasize is the absence of multispecies coalescent (MSC) approaches such as BPP [76], STACEY [85], or BEAST [86]. These methods explicitly model the stochastic processes of lineage sorting and can accommodate gene tree heterogeneity across multiple unlinked nuclear loci. Our reliance on concatenated mtDNA means that we cannot confidently distinguish between population structure, recent divergence, and true species boundaries. Consequently, the taxonomic suggestions offered below (e.g., potential synonymy among certain taxa) should be viewed as hypotheses requiring independent testing with nuclear markers (e.g., RAD-seq, exon capture) and MSC-based delimitation. With these caveats, we note that certain species pairs show consistently low genetic distances (<2% K2P for *Cytb*), lack reciprocal monophyly, and exhibit overlapping meristic ranges. These include: *L. capito* with *L. conocephalus*; *L. esocinus* with *L. xanthopterus*; *L. barbulus* with *L. kersin*; *B. lacerta* with *B. cyri*; and *B. lacerta* with *B. karunensis*. While these findings raise the possibility of synonymy, we stop short of formal taxonomic revision pending MSC analyses with nuclear data. Similarly, the close affinity among *Arabibarbus*, *Mesopotamichthys*, and *Carasobarbus* (genetic distances 4–6%, below the 8% threshold suggested by Roul et al., [71] for generic separation) suggests that their generic status warrants re-examination, but again, we present this as a hypothesis rather than a conclusion.

Based on our analyses of genetic distances, phylogeny of two genes, and haplotype networks, we have identified three distinct evolutionary entities within the Barbels group. However, our findings also strongly suggest the presence of cryptic diversity or undescribed species, which we propose as hypotheses for further investigation. Specifically, the results indicate that several lineages warrant further taxonomic scrutiny. The KH40 specimen, which showed a significant genetic distance of 7% from *C. sublimus* and was placed in a separate clade, suggests the potential for a new species. Similarly, the lineage comprising samples HK05, HK42, and HK51 was found in *L. barbulus*, indicating they may represent a different, undescribed species. The separate placement of the HK46/HK47 (*B. lacerta*) and HK55 (*B. cyri*) samples, with genetic distances of 4.6% and 2.8% respectively, further supports the hypothesis of cryptic species within the Barbus (str) group. While we do not formally describe these as new species at this time, the evidence from our molecular analyses including significant genetic distances and distinct phylogenetic placement presents compelling hypotheses for their existence. Future research incorporating additional molecular markers (e.g., nuclear genes) and morphological data is essential to confirm the taxonomic status of these lineages. Additionally, our finding that *Carasobarbus*, *Arabibarbus*, and *Mesopotamichthys* may not belong to the Barbels taxa provides a hypothesis for further investigation.

### Evolutionary history of Iranian Barbels

The estimated divergence within the Barbels taxa began during the Oligocene (median = 34.3 Mya; 95% HPD: 26.8–40.7 Mya). This period coincides broadly with the closure and subduction of the Neo-Tethys oceanic crust beneath the Iranian plate and the initial formation of the Zagros fold-thrust belt [87–89]. However, given the wide credible intervals around our divergence estimates and the reliance on a single secondary calibration, we cannot definitively establish a causal link. Instead, we suggest that these orogenic events may have contributed to the allopatric isolation of ancestral lineages, but alternative drivers (e.g., eustatic sea-level changes, climatic oscillations) cannot be excluded.

Another divergence was estimated at approximately 21.1 Mya (95% HPD: 18.6–26.1 Mya), which falls within the period of continental collision and early Zagros orogeny. This temporal overlap raises the possibility that mountain building contributed to lineage divergence, but this interpretation remains speculative given the uncertainties in our dating. At this time, the divergence between the clades *Luciobarbus* and *Barbus* (str) occurred. This divergence timeframe aligns with the findings of [74,90,91].

During this period, the formation of mountain ranges from the Alps to the Himalayas, due to continental collisions, is thought to have played a role in the structural shaping of the Zagros Mountains, the Mesopotamian region, and the Persian Gulf [92,93]. The two subsequent divergence events within the central part of the Barbels group lineage occurred in the early Miocene and mid-Miocene (12.3; 95% HPD: 8.1–13.9 Mya and 11.4; 95% HPD: 7.5–14.7 Mya). This era was marked by significant climatic and tectonic shifts that may have influenced the speciation process [94–97]. The collision of the Indo-Asia and Arabian plates from the Miocene onward is considered a significant factor that could have acted as a physical barrier, potentially leading to adaptation, evolutionary divergence, and reproductive isolation among some native species [98,99]. Geological evidence suggests that the Zagros Mountains underwent substantial uplift (Outer Zagros), which could have been instrumental in the creation of new basins and the emergence of new species. [92,100]. As also noted by Ahmadzadeh et al. [101] and Krause [102].

According to the dating tree, some species in the *Luciobarbus* and *Barbus* (sl) clade have a separation time of less than two million years, in some cases under one million. This is particularly evident within the Barbus clade (Cl 2) (Fig 6). As Regalado et al. [103] suggest, caution is warranted when interpreting these findings for complex and cryptic species that have recently undergone speciation events (less than 2 Mya). Due to the short time frame, their morphological and other distinguishing characteristics may not have fully developed. Therefore, further study and examination of additional specimens from this lineage are needed for a more comprehensive understanding. It’s important to note that no specific geological changes have been documented that would explain the short-term distribution range of these recently diverged species. An alternative hypothesis is that they may be the result of a single lineage with two distinct veins. For instance, *L. esocinus* is estimated to have diverged from *L. xanthopterus* at about 800 Kya (95% HDP: 400 Kya-1.2 Mya). The close genetic resemblance between these species, as concluded by Faddagh et al. (2012) [104]  based on nuclear data, is consistent with this idea. The debate over whether these should be considered a single species or two separate ones, as noted by Karaman [41], Fricke et al. [105] and Almaça [106] and Kuru [107] highlights the complexities.

Additionally, the *Barbus* s.str species (Cl 2) are known to be young, diverging approximately from the Pleistocene onwards (under 2.5 Mya). Despite their close genetic and morphological affinity, they are found in different basins. One hypothesis is that these species may still experience gene flow through a process such as headwater stream capture, as suggested by Khaefi et al. (2017). This mechanism could facilitate the introduction of species from one basin to another. The proximity between the headwaters of different basins likely plays a significant role in the potential spread of species. For example, the close genetic relationship between *B. miliaris* and *B. cyri* could be attributed to the nearness of several Salt Lake Rivers in the north to the southern Caspian Sea basin. Similarly, the close association between *B. miliaris* and *B. lacerta* is likely due to the proximity of western rivers in the salt lake basin to the eastern origins of the Tigris River drainage.

We acknowledge that relying on a single secondary calibration point is a significant limitation of this study. Secondary calibrations propagate errors from the original study and lack the independent validation provided by primary fossil calibrations [108,109]. Without multiple independent calibration points (e.g., from well-dated fossils within the ingroup or from multiple nuclear genes), the absolute timeline presented here remains provisional. Therefore, while our analyses suggest a temporal correspondence with the Zagros uplift and Neo-Tethys closure, these interpretations are hypothesis-generating rather than conclusive. Future studies incorporating fossil cyprinids from the Middle East and additional molecular markers are needed to refine these estimates.

### Ecological Niche evolution

We investigated the interaction between the evolutionary history of Barbels group species and their ecological niches, aiming to understand how climate variables may have influenced their evolution. Our analysis revealed notable differences and similarities in the climate tolerances of closely related species within the group.

While several species pairs (e.g., *L. esocinus*–*L. xanthopterus*) show high raw niche overlap (D > 0.85), equivalency tests indicate that their niches are not statistically identical. However, similarity tests suggest that observed overlaps generally do not exceed random expectations given the background environments. This pattern implies that climatic niche differences among Iranian Barbel lineages are largely explained by allopatric geographic separation (i.e., occupancy of different basins with distinct climates) rather than by evolutionary niche divergence driven by ecological specialization. Notably, *L. mursa* and *B. miliaris* exhibit the lowest niche overlap with congeners, consistent with their restricted distributions in high-altitude headwaters and the Namak Lake endorheic basin, respectively. For these species, local adaptation to extreme climatic conditions (e.g., low minimum temperatures, low annual precipitation) may have played a role in their diversification, but our data cannot distinguish between adaptation and dispersal limitation. Cautionary note: Our statistical tests have several limitations. First, small sample sizes for rare species (e.g., *C. kosswigi*, n = 3 occurrences) precluded formal modeling, and these species were excluded from pairwise comparisons. Second, niche equivalency tests are known to be sensitive to spatial biases in occurrence data. Third, our models do not account for non-climatic factors (e.g., hydrological connectivity, water chemistry, biotic interactions) that may be equally or more important for Barbel distribution. While climate can influence speciation by causing population isolation, it is important to recognise that observing niche evolution within a clade does not definitively confirm speciation or rule out other possibilities, such as niche conservatism or divergence [110–112]. A thorough examination of the evolutionary history of Barbels group species across five bioclimatic variables revealed fascinating patterns of niche dynamics. For example, the *Carasobarbus* and *Arabibarbus* clades exhibited similar patterns in temperature and precipitation variables, suggesting they have similar ecological tolerances. This is particularly evident between the *Arabibarbus* and *Mesopotamichthys* clades, which share identical climatic niches across most variables, especially given their distribution in the western and southwestern parts of Iran. This convergence of niches suggests that these groups may have evolved in response to similar environmental conditions during their evolutionary history. The *Luciobarbus* clade displayed more complex patterns. For instance, species from the northern regions of the country showed different niches compared to those from the southern regions. We observed that *L. barbulus*, *L. esocinus*, *L. xanthopterus*, and *L. kersin* have overlapping ranges in all five climate factors. This overlap likely reflects shared habitat preferences, a common trait among geographically separated populations. Similarly, the convergence of climatic habitats for *L. conocephalus*, *L. mursa*, *L. brachycephalus*, and *L. capito* suggests shared ecological adaptations despite their distinct geographic basins. For the Barbus (str) clade, *B. lacerta*, *B. cyri*, and *B. karunensis* exhibited similar niches, while *B. miliaris* showed distinct climatic niche patterns. However, it is crucial to note that the limited number of species occurrence points, as indicated by Ahmadi et al. [113] and Ahmadzadeh et al. [114], can introduce uncertainty into these niche evolution analyses. The precipitation of the driest month showed the most robust phylogenetic signal, suggesting it may be a significant variable influencing the distribution and speciation of the Barbels group. Therefore, while we find limited evidence for climatic niche divergence as a primary driver of speciation in this group, we cannot rule out its role in combination with other mechanisms. Future studies incorporating genome-wide data and physiological experiments will be necessary to test whether the subtle climatic differences detected here translate into meaningful ecological specialization or reproductive isolation.

### Morphology

The impact of environmental factors on meristic characters suggests that these characteristics can indicate the presence of geographic separation between populations during early life stages, offering valuable insights for identifying different population components [115–117]. The results of the PCA and CVA plots displayed that for the two plots, the *Luciobarbus* and *Barbus* (str) genera are together in a group, and the PCA plot of the other genera containing *Arabibarbus*, *Mesopotamichthys,* and *Carasobarbus* were in another group. On the other hand, CVA for these three genera showed that *Arabibarbus* and *Mesopotamichthys* are separate from the *Carasobarbus* genus. Considering that the environment does not influence the meristic traits and is dependent on the individual’s genetics, the grouping of *Luciobarbus* with the *Barbus* (str) could show the low genetic difference between the species of these genera. In contrast, the separation of these two genera from the three genera of *Arabibarbus*, *Mesopotamichthys,* and *Carasobarbus* can also indicate genetic differences between these three genera, confirming our phylogeny analysis results. Turan et al. [118] believed that fishes during their larval development in similar environmental conditions often have identical numerical characteristics during adulthood. Hence, it is likely that the genera that are in the same group, their species experience similar larval conditions in terms of physical and chemical characteristics. The current study depicted that the most effective meristics characters on Barbels species were Dorsal fin branched rays, pectoral fin branched rays, and Lateral line scales. According to our result, the Barbels group species’ most effective meristics characteristics were dorsal fin branched rays, pectoral fin branched rays, and lateral line scales. This result was in line with Krpo-Ćetković and Stamenković [119], who concluded Dorsal fin branched rays and Lateral line scales were considered the most critical meristic features in the separation of fish. The findings from the meristic examination of Barbels group species partially shed light on the distinctions among the genera. However, further sampling is necessary to draw definitive conclusions and establish more robust evidence. Combining meristic analysis with other methods yields the most effective results. In addition, the samples captured in the present study did not have significant meristic differences compared to other similar species in previous studies.

## Conclusion

We investigated the Iranian Barbels taxon using an integrative approach that incorporated phylogenetic relationships, ecological niches, and morphology. While our results raise the possibility that several named species (e.g., *L. capito* with *L. conocephalus*; *L. esocinus* with *L. xanthopterus*; *L. barbulus* with *L. kersin*; *B. lacerta* with *B. cyri*; *B. lacerta* with *B. karunensis*) and genera (*Arabibarbus* with *Mesopotamichthys*) may be synonymous, we present these as testable hypotheses rather than formal taxonomic conclusions. The discrepancy between GMYC and bPTP (22 vs. 14 clusters), the absence of multispecies coalescent methods, and the reliance on mtDNA alone preclude definitive taxonomic revision at this stage. We therefore recommend that future studies employ nuclear phylogenomics and MSC-based delimitation to resolve species boundaries in this morphologically challenging group.

## Supporting information

S1 Table List of species, Locality and GenBank accession numbers separated according to applied genes. (PDF)

S2 Table Characteristics related to meristic traits of species of Barbels group. (PDF)

S3 Table Location of Barbels speceis for ecological niche evolution. (PDF)

S4 Table Cross-validation of divergence time estimates (Mya) for key nodes within the Iranian Barbels group under different calibration strategies. (PDF)

S5 Table PCA Results (Standardized Data). (PDF)

S6 Table Summary of ecological niche model (MaxEnt) performance and variable contributions for Iranian Barbels taxon. Values shown are means ± standard deviation (SD) across 10 bootstrap replicates. Only species with ≥5 unique occurrence localities were modeled. BIO1 = Annual Mean Temperature, BIO7 = Temperature Annual Range, BIO14 = Precipitation of Driest Month. AUC = Area Under the Curve; TSS = True Skill Statistic. Dashes (—) indicate that the species was excluded due to insufficient sample size.
(PDF)

S7 Table Statical niche overlap values; Schoener’s D, Hellinger’s and Range for Iranian Barbels taxon species. (PDF)

S8 Fig Lineage Through Time Plot: number of candidate species for Barbels. The raw axis is time in millions of years.
(PDF)

S9 Data COI sequences used from NCBI. (FAS)

S10 Data *Cytb* sequences used from NCBI. (FAS)

S11 Data The *Cytb* uncorrected genetic distances. (XLS)

S12 Data The COI uncorrected genetic distances. (XLS)

## References

[1] MacDonald GM. Biogeography: Introduction to Space, Time, and Life. John Wiley & Sons; 2025.

[2] Bagheri M, Azimi M, Khoshnamvand H, Abdoli A, Ahmadzadeh F. The threat of a non-native oligochaete species in Iran’s freshwater: assessment of the diversity and origin of Eiseniella tetraedra (Savigny, 1826) and its response to climate change. Biol Open. 2023:bio.060180. doi: 10.1242/bio.060180 PMC10840848 38014991

[3] Khoshnamvand H, Malekian M, Keivani Y, Goudarzi F. DNA barcoding of the Luristan newt (Neurergus kaiseri) in south-western Iran. J Wildl Biodivers. 2019;3(2). doi: 10.22120/jwb.2019.34933

[4] Makki T, Mostafavi H, Matkan AA, Valavi R, Hughes RM, Shadloo S, et al. Predicting climate heating impacts on riverine fish species diversity in a biodiversity hotspot region. Sci Rep. 2023;13(1):14347. doi: 10.1038/s41598-023-41406-9 37658153 PMC10474041

[5] Smith SD, Pennell MW, Dunn CW, Edwards SV. Phylogenetics is the New Genetics (for Most of Biodiversity). Trends Ecol Evol. 2020;35(5):415–25. doi: 10.1016/j.tree.2020.01.005 32294423

[6] Williams DM, Wheeler QD. The New Taxonomy: A Science Reimagined. CRC Press. 2025.

[7] Mostafavi H, Pletterbauer F, Coad BW, Mahini AS, Schinegger R, Unfer G, et al. Predicting presence and absence of trout (Salmo trutta) in Iran. Limnologica. 2014;46:1–8. doi: 10.1016/j.limno.2013.12.001 24707064 PMC3974070

[8] Mostafavi H, Schinegger R, Melcher A, Moder K, Mielach C, Schmutz S. A new fish-based multi-metric assessment index for cyprinid streams in the Iranian Caspian Sea Basin. Limnologica. 2015;51:37–52. doi: 10.1016/j.limno.2014.10.006 25960581 PMC4418740

[9] Zachos FE. (New) Species concepts, species delimitation and the inherent limitations of taxonomy. J Genet. 2018;97(4):811–5. doi: 10.1007/s12041-018-0965-1 30262692

[10] Hending D. Cryptic species conservation: a review. Biol Rev Camb Philos Soc. 2025;100(1):258–74. doi: 10.1111/brv.13139 39234845 PMC11718601

[11] Malekian M. Morphological assessment raises the possibility of cryptic species within the Luristan newt, Neurergus kaiseri (Amphibia: Salamandridae). HJ. 2019;29(4):237–44. doi: 10.33256/hj29.4.237244

[12] Rheindt FE, Bouchard P, Pyle RL, Welter-Schultes F, Aescht E, Ahyong ST, et al. Tightening the requirements for species diagnoses would help integrate DNA-based descriptions in taxonomic practice. PLoS Biol. 2023;21(8):e3002251. doi: 10.1371/journal.pbio.3002251 37607211 PMC10443861

[13] Antil S, Abraham JS, Sripoorna S, Maurya S, Dagar J, Makhija S, et al. DNA barcoding, an effective tool for species identification: a review. Mol Biol Rep. 2023;50(1):761–75. doi: 10.1007/s11033-022-08015-7 36308581

[14] Chang H, Ye T, Xie Z, Liu X. Application of environmental DNA in aquatic ecosystem monitoring: opportunities, challenges and prospects. Water. 2025;17(5):661. doi: 10.3390/w17050661

[15] Yao Y, Chen J-Y, Gong X-L, Li C-H, Liu Z, Lin X-L. Species Delimitation and Cryptic Diversity in Rheotanytarsus Thienemann & Bause, 1913 (Diptera: Chironomidae) Based on DNA Barcoding. Insects. 2025;16(4):370. doi: 10.3390/insects16040370 40332883 PMC12028281

[16] Hu Y, Fan H, Chen Y, Chang J, Zhan X, Wu H, et al. Spatial patterns and conservation of genetic and phylogenetic diversity of wildlife in China. Sci Adv. 2021;7(4):eabd5725. doi: 10.1126/sciadv.abd5725 33523945 PMC10671236

[17] Teles JN, Mantelatto FL. Tracking genetic and phylogenetic diversity across Brazilian ecoregions: a molecular ecology approach using marine decapod crustaceans. J Crustac Biol. 2024;44(3):ruae057. doi: 10.1093/jcbiol/ruae057

[18] Ferrari C, Marelli SP, Bagnato A, Cerolini S, Strillacci MG. Sequencing and characterization of complete mitogenome DNA of worldwide turkey (Meleagris gallopavo) populations. Animal Biotechnology. 2024;35(1):2397682. doi: 10.1080/10495398.2024.2397682 39262293 PMC12674428

[19] Piccoli C, Scrima R, D’Aprile A, Chetta M, Cela O, Pacelli C, et al. Pathogenic DNM1L Variant (1085G>A) Linked to Infantile Progressive Neurological Disorder: Evidence of Maternal Transmission by Germline Mosaicism and Influence of a Contemporary in cis Variant (1535T>C). Genes. 2021;12(9):1295. doi: 10.3390/genes12091295 34573276 PMC8467311

[20] Sayyadzadeh G, Esmaeili HR. Freshwater lamprey and fishes of Iran: reappraisal and updated checklist with a note on Eagderi *et al*. (2022). Zootaxa. 2024;5402(1):Art. no. 1. doi: 10.11646/zootaxa.5402.1.1 38480458

[21] Eagderi S, Moulodi-saleh A, Esmaeili HR, Sayyadzadeh G, Nasri M. Freshwater lamprey and fishes of Iran; a revised and updated annotated checklist-2022. Turk J Zool. 2022;46(6):500–22. doi: 10.55730/1300-0179.3104

[22] Çiçek E, et al. Freshwater lampreys and fishes in the Middle East. TAXA. 2024;4.

[23] Khoshnamvand H, et al. A different destiny after the ice age: impacts of climate change on the global biogeography of Carasobarbus. Environ Sustain Indic. 2025;26:100646. doi: 10.1016/j.indic.2025.100646

[24] Khoshnamvand H, Mousavi SM, Darvishi A, Ahmadi K, Naghibi A, Janko K, et al. Macroecological predictors to determine future refuges of Luciobarbus species in the Tigris–Euphrates basin: rethinking conservation strategies and management. Global Ecology and Conservation. 2025;57:e03394. doi: 10.1016/j.gecco.2024.e03394

[25] Nelson JS, Grande TC, Wilson MVH. Fishes of the World. 1st ed. Wiley. 2016. doi: 10.1002/9781119174844

[26] Makki T, Mostafavi H, Matkan A, Aghighi H. Modelling climate-change impact on the spatial distribution of Garra Rufa (Heckel, 1843) (Teleostei: Cyprinidae). Iran J Sci Technol Trans Sci. 2021;45(3):795–804. doi: 10.1007/s40995-021-01088-2

[27] Yang L, Naylor GJP, Mayden RL. Deciphering reticulate evolution of the largest group of polyploid vertebrates, the subfamily cyprininae (Teleostei: Cypriniformes). Mol Phylogenet Evol. 2022;166:107323. doi: 10.1016/j.ympev.2021.107323 34634450

[28] Borkenhagen K. A new genus and species of cyprinid fish (Actinopterygii, Cyprinidae) from the Arabian Peninsula, and its phylogenetic and zoogeographic affinities. Environ Biol Fish. 2014;97(10):1179–95. doi: 10.1007/s10641-014-0315-y

[29] Borkenhagen K. Molecular phylogeny of the tribe Torini Karaman, 1971 (Actinopterygii: Cypriniformes) from the Middle East and North Africa. Zootaxa. 2017;4236(2):zootaxa.4236.2.4. doi: 10.11646/zootaxa.4236.2.4 28264326

[30] Borkenhagen K, Esmaeili HR, Mohsenzadeh S, Shahryari F, Gholamifard A. The molecular systematics of the Carasobarbus species from Iran and adjacent areas, with comments on Carasobarbus albus (Heckel, 1843). Environ Biol Fishes. 2011;91(3):327–35. doi: 10.1007/s10641-011-9787-1

[31] Coad BW. Freshwater fishes of Iran. Canadian Museum of Nature Ottawa. 1992;66.

[32] Coad BW. Systematic biodiversity in the freshwater fishes of Iran. Ital J Zool. 1998;65(sup1):101–8. doi: 10.1080/11250009809386802

[33] Coad BW. Endemicity in the Freshwater Fishes of Iran. Iran J Anim Biosyst. 2005;1(1). doi: 10.22067/ijab.v1i1.36733

[34] Eagderi S, Nikmehr N, Í‡i í§ek E, Esmaeili HR, Vatandoust S, Mousavi-Sabet H. Barbus urmianus a new species from Urmia Lake basin, Iran (Teleostei: Cyprinidae). Int J Aquat Biol. 2019;7(4):Art. no. 4. doi: 10.22034/ijab.v7i4.725

[35] Esmaeili HR, Coad BW, Gholamifard A, Nazari N, Teimory A. Annotated checklist of the freshwater fishes of Iran. Zoosystematica Ross. 2010;19(2):361–86. doi: 10.31610/zsr/2010.19.2.361

[36] Jouladeh-Roudbar A. Distribution and taxonomy of the Barbus Cuvier and Cloquet, 1816 in Iran using COI gene. MGJ. 2021;16(2):125–32.

[37] Khaefi R, Teimori A, Esmaeili HR. Phylogenetic relationships and taxonomy of Luciobarbus barbulus (Heckel, 1847) (Teleostei: Cyprinidae). J Ichthyol. 2017;57(6):835–45. doi: 10.1134/S0032945217060078

[38] Khaefi R, Vatandoust S, Esmaeili HR. Redescription of Barbus miliaris de Filippi, 1863 (Teleostei: Cyprinidae) from the endorheic Namak Lake basin of Iran. FishTaxa. 2017;2:33–42.

[39] Mostafavi H, Mehrabian AR, Teimori A, Shafizade-Moghadam H, Kambouzia J. The ecology and modelling of the freshwater ecosystems in Iran. In: Jawad LA, editor. Tigris and Euphrates Rivers: their environment from headwaters to mouth. Cham: Springer International Publishing; 2021. p. 1143–200. doi: 10.1007/978-3-030-57570-0_52

[40] Motamedi M, Madjdzadeh SM, Teimori A, Esmaeili HR, Mohsenzadeh S. Morphological and molecular perspective on geographical differentiation of Barbus populations (Actinopterygii; Cyprinidae) within Iranian freshwater drainages. Turk J Fish Aquat Sci. 2014;14(2). doi: 10.4194/1303-2712-v14_2_05

[41] Karaman M. Revision der Barben Europas, Vorderasiens und Nordafricas. Susswasserfische der Turkei. 1971;67:175–274.

[42] Valiallahi J. Comparison of two subspecies of Barbus capito in southern parts of Caspian Sea basin. Taxon Biosyst. 2010;2(3):67–77.

[43] Abdoli A. *Field guide of fishes of inland waters of Iran*, First. Tehran: Iran-shenasi; 2016.

[44] Sambrook J, Fritsch EF, Maniatis T. Molecular cloning: a laboratory manual. Cold Spring Harb. Lab. Press; 1989.

[45] Katoh K, Rozewicki J, Yamada KD. MAFFT online service: multiple sequence alignment, interactive sequence choice and visualization. Brief Bioinform. 2019;20(4):1160–6. doi: 10.1093/bib/bbx108 28968734 PMC6781576

[46] Akaike H. A new look at the statistical model identification. IEEE Trans Automat Contr. 1974;19(6):716–23. doi: 10.1109/tac.1974.1100705

[47] Nguyen L-T, Schmidt HA, von Haeseler A, Minh BQ. IQ-TREE: a fast and effective stochastic algorithm for estimating maximum-likelihood phylogenies. Mol Biol Evol. 2015;32(1):268–74. doi: 10.1093/molbev/msu300 25371430 PMC4271533

[48] Hoang DT, Chernomor O, von Haeseler A, Minh BQ, Vinh LS. UFBoot2: improving the ultrafast bootstrap approximation. Mol Biol Evol. 2018;35(2):518–22. doi: 10.1093/molbev/msx281 29077904 PMC5850222

[49] Huelsenbeck JP, Ronquist F. MRBAYES: Bayesian inference of phylogenetic trees. Bioinformatics. 2001;17(8):754–5. doi: 10.1093/bioinformatics/17.8.754 11524383

[50] Rambaut A, Drummond AJ, Xie D, Baele G, Suchard MA. Posterior summarization in bayesian phylogenetics using tracer 1.7. Syst Biol. 2018;67(5):901–4. doi: 10.1093/sysbio/syy032 29718447 PMC6101584

[51] Anisimova M, Gil M, Dufayard J-F, Dessimoz C, Gascuel O. Survey of branch support methods demonstrates accuracy, power, and robustness of fast likelihood-based approximation schemes. Syst Biol. 2011;60(5):685–99. doi: 10.1093/sysbio/syr041 21540409 PMC3158332

[52] Shimodaira H, Hasegawa M. Multiple comparisons of log-likelihoods with applications to phylogenetic inference. Molecular Biology and Evolution. 1999;16(8):1114. doi: 10.1093/oxfordjournals.molbev.a026201

[53] Kumar S, Stecher G, Li M, Knyaz C, Tamura K. MEGA X: molecular evolutionary genetics analysis across computing platforms. Mol Biol Evol. 2018;35(6):1547–9. doi: 10.1093/molbev/msy096 29722887 PMC5967553

[54] Bandelt HJ, Forster PF, Rohl A. Median-joining networks for inferring intraspecific phylogenies. Mol Biol Evol. 1999;16(1):37–48. doi: 10.1093/oxfordjournals.molbev.a026036 10331250

[55] Pons J, Barraclough TG, Gomez-Zurita J, Cardoso A, Duran DP, Hazell S, et al. Sequence-based species delimitation for the DNA taxonomy of undescribed insects. Syst Biol. 2006;55(4):595–609. doi: 10.1080/10635150600852011 16967577

[56] Zhang J, Kapli P, Pavlidis P, Stamatakis A. A general species delimitation method with applications to phylogenetic placements. Bioinformatics. 2013;29(22):2869–76. doi: 10.1093/bioinformatics/btt499 23990417 PMC3810850

[57] Drummond AJ, Rambaut A. BEAST: Bayesian evolutionary analysis by sampling trees. BMC Evol Biol. 2007;7:214. doi: 10.1186/1471-2148-7-214 17996036 PMC2247476

[58] Levin BA, Freyhof J, Lajbner Z, Perea S, Abdoli A, Gaffaroğlu M, et al. Phylogenetic relationships of the algae scraping cyprinid genus Capoeta (Teleostei: Cyprinidae). Mol Phylogenet Evol. 2012;62(1):542–9. doi: 10.1016/j.ympev.2011.09.004 21967785

[59] Levin BA, Gandlin AA, Simonov ES, Levina MA, Barmintseva AE, Japoshvili B, et al. Phylogeny, phylogeography and hybridization of Caucasian barbels of the genus Barbus (Actinopterygii, Cyprinidae). Mol Phylogenet Evol. 2019;135:31–44. doi: 10.1016/j.ympev.2019.02.025 30844445

[60] Ho SY, Duchêne S. Molecular‐clock methods for estimating evolutionary rates and timescales. Molecular ecology. 2014;23(24):5947-65. doi: 10.1111/mec.12953 25290107

[61] Wang J, et al. Molecular phylogeny of European and African Barbus and their West Asian relatives in the Cyprininae (Teleostei: Cypriniformes) and orogenesis of the Qinghai-Tibetan Plateau. Chinese Science Bulletin. 2013;58(31):3738–46. doi: 10.1007/s11434-013-5878-z

[62] Talwar W, Jhingran J. Inland fishes of India and adjacent countries: P K Talwar and A G Jhingran (eds) A A Balkema, Rotterdam, The Netherlands. Rev Fish Biol Fish. 1994;4(1):135–6. doi: 10.1007/BF00043269

[63] Nelson R. Evolutionary social science and universal Darwinism. J Evol Econ. 2006;16:491–510. doi: 10.1007/s00191-006-0025-5

[64] Coad BW. Carps and minnows of Iran (families cyprinidae and leuciscidae). Vol. I: General introduction and carps (family cyprinidae). Not specified: Not specified; 2021.

[65] R Development Core Team. R: A Language and Environment for Statistical Computing; 2021.

[66] Heibl C, Calenge C. Phyloclim: integrating phylogenetics and climatic niche modeling. 0.9.5. 2009. 10.32614/CRAN.package.phyloclim

[67] Lohse K. Can mtDNA Barcodes Be Used to Delimit Species? A Response to Pons *et al*. (2006). Systematic Biology. 2009;58(4):439–42. doi: 10.1093/sysbio/syp039 20525596

[68] Luo A, Ling C, Ho SY, Zhu CD. Comparison of methods for molecular species delimitation across a range of speciation scenarios. Systematic Biology. 2018;67(5):830-46. doi: 10.1093/sysbio/syy011 29462495 PMC6101526

[69] Toews DP, Brelsford A. The biogeography of mitochondrial and nuclear discordance in animals. Molecular ecology. 2012;21(16):3907-30. doi: 10.1111/j.1365-294X.2012.05664.x 22738314

[70] Bagley JC, Alda F, Breitman MF, Bermingham E, van den Berghe EP, Johnson JB. Assessing species boundaries using multilocus species delimitation in a morphologically conserved group of neotropical freshwater fishes, the Poecilia sphenops species complex (Poeciliidae). PLoS One. 2015;10(4):e0121139. doi: 10.1371/journal.pone.0121139 PMC4388586 25849959

[71] Roul SK, Jeena NS, Kumar R, Vinothkumar R, Rahangdale S, Rahuman S, et al. Postulating the Modality of Integrative Taxonomy in Describing the Cryptic Congener Pampus griseus (Cuvier) and Systematics of the Genus Pampus (Perciformes: Stromateidae). Front Mar Sci. 2021;8. doi: 10.3389/fmars.2021.778422

[72] Galván-Quesada S, Doadrio I, Alda F, Perdices A, Reina RG, García Varela M, et al. Molecular Phylogeny and Biogeography of the Amphidromous Fish Genus Dormitator Gill 1861 (Teleostei: Eleotridae). PLoS One. 2016;11(4):e0153538. doi: 10.1371/journal.pone.0153538 27074006 PMC4830628

[73] Faddagh MS, Najah H, Issa A-B. DNA fingerprinting of eight cyprinid fish species of Iraqi inland waters using RAPD-PCR technique.

[74] Durand J-D, Tsigenopoulos CS, Unlü E, Berrebi P. Phylogeny and biogeography of the family Cyprinidae in the Middle East inferred from cytochrome b DNA- evolutionary significance of this region. Mol Phylogenet Evol. 2002;22(1):91–100. doi: 10.1006/mpev.2001.1040 11796032

[75] Tsigenopoulos CS, Kasapidis P, Berrebi P. Phylogenetic relationships of hexaploid large-sized barbs (genus Labeobarbus, Cyprinidae) based on mtDNA data. Mol Phylogenet Evol. 2010;56(2):851–6. doi: 10.1016/j.ympev.2010.02.006 20152918

[76] Yang L, Sado T, Vincent Hirt M, Pasco-Viel E, Arunachalam M, Li J, et al. Phylogeny and polyploidy: resolving the classification of cyprinine fishes (Teleostei: Cypriniformes). Mol Phylogenet Evol. 2015;85:97–116. doi: 10.1016/j.ympev.2015.01.014 25698355

[77] Jouladeh-Roudbar A, Ghanavi HR, Doadrio I. Ichthyofauna from Iranian freshwater: annotated checklist, diagnosis, taxonomy, distribution and conservation assessment. Zool Stud. 2020;59. doi: 10.6620/ZS.2020.59-21 PMC7807176 33456548

[78] Khaefi R, Esmaeili HR, Eagderi S, Geiger M. Taxonomic review of the cryptic Barbus lacerta species group with description of a new species (Teleostei: Cyprinidae). FishTaxa. 2017;2:90–115.

[79] Beltrán-López RG, Domínguez-Domínguez O, Guerrero JA, Corona-Santiago DK, Mejía-Mojica H, Doadrio I. Phylogeny and taxonomy of the genus Ilyodon Eigenmann, 1907 (Teleostei: Goodeidae), based on mitochondrial and nuclear DNA sequences. J Zool Syst Evol Res. 2017;55(4):340–55. doi: 10.1111/jzs.12175

[80] Jouladeh-Roudbar A, Farahmand H, Abed Elmdoust A, Mojazi Amiri B, Eagderi S. Study on phylogenetic status of Hari barbel Luciobarbus conocephalus (Kessler, 1872) from Hari river using Cytb gene. J Aquat Ecol. 2022;11(4):21–9.

[81] Parmaksız A, Korkmaz E, Ulusal D, Doğan N. Phylogenetic analysis of Luciobarbus Heckel, 1843 and Barbus Cuvier & Cloquet, 1816 species in the Euphrates River (Turkey) based on mtDNA COI gene sequences. Aquat Res. 2022;5(2):129–35. doi: 10.3153/AR22012

[82] Abasi Dehkord I, Hashemzadeh Segherloo I, Poria M, Khajeh P. Analysis of phylogenetic status of Luciobarbus esocinus, Luciobarbus xanthopterus, Tor grypus, and Mesopotamichties sharpeyi. J Fish. 2018;71(2). doi: 10.22059/jfisheries.2018.242522.999

[83] Carstens BC, Pelletier TA, Reid NM, Satler JD. How to fail at species delimitation. Molecular ecology. 2013;22(17):4369-83. doi: 10.1111/mec.12413 23855767

[84] Sukumaran J, Knowles LL. Multispecies coalescent delimits structure, not species. Proceedings of the National Academy of Sciences. 2017;114(7):1607-12. doi: 10.1073/pnas.1607921114 PMC5320999 28137871

[85] Jones OP, Voets NL, Adcock JE, Stacey R, Jbabdi S. Resting connectivity predicts task activation in pre-surgical populations. NeuroImage: Clinical. 2017;13:378-85. doi: 10.1016/j.nicl.2016.12.028 28123949 PMC5222953

[86] Heled J, Drummond AJ. Bayesian inference of species trees from multilocus data. Molecular biology and evolution. 2009;27(3):570-80. doi: 10.1093/molbev/msp274 19906793 PMC2822290

[87] Berberian F, Muir ID, Pankhurst RJ, Berberian M. Late Cretaceous and early Miocene Andean-type plutonic activity in northern Makran and Central Iran. J Geol Soc. 1982;139(5):605–14. doi: 10.1144/gsjgs.139.5.0605

[88] Homke S, Vergés J, Serra-Kiel J, Bernaola G, Sharp I, Garcés M, et al. Late Cretaceous–Paleocene formation of the proto–Zagros foreland basin, Lurestan Province, SW Iran. GSA Bulletin. 2009;121(7–8):963–78. doi: 10.1130/b26035.1

[89] Mohajjel M, Fergusson CL. Dextral transpression in Late Cretaceous continental collision, Sanandaj–Sirjan Zone, western Iran. Journal of Structural Geology. 2000;22(8):1125–39. doi: 10.1016/s0191-8141(00)00023-7

[90] Machordom A, Doadrio I. Evidence of a cenozoic Betic-Kabilian connection based on freshwater fish phylogeography (Luciobarbus, Cyprinidae). Mol Phylogenet Evol. 2001;18(2):252–63. doi: 10.1006/mpev.2000.0876 11161760

[91] Zardoya R, Doadrio I. Molecular evidence on the evolutionary and biogeographical patterns of European cyprinids. J Mol Evol. 1999;49(2):227–37. doi: 10.1007/pl00006545 10441674

[92] Ajirlu MS, Moazzen M, Hajialioghli R. Tectonic evolution of the Zagros Orogen in the realm of the Neotethys between the Central Iran and Arabian Plates: an ophiolite perspective. Central European Geology. 2016;59(1–4):1–27. doi: 10.1556/24.59.2016.001

[93] Khoshnamvand H, Azimi M, Ahmadzadeh F, Abdoli A, Janko K. Integrating historical biogeography and Pliocene climate fluctuation to unravel the evolution of Tigris-Euphrates drainage basin through widespread freshwater Barbinae (Cypriniformes: Cyprinidae). Inland Waters. 2025;15(1):2455205. doi: 10.1080/20442041.2025.2455205

[94] Ahmadzadeh F, Lymberakis P, Pirouz RS, Kapli P. The evolutionary history of two lizards (Squamata: Lacertidae) is linked to the geological development of Iran. Zool Anz. 2017;270:49–56. doi: 10.1016/j.jcz.2017.09.003

[95] Ghaedi Z, Badri S, Saberi-Pirooz R, Vaissi S, Javidkar M, Ahmadzadeh F. The Zagros Mountains acting as a natural barrier to gene flow in the Middle East: more evidence from the evolutionary history of spiny-tailed lizards (Uromasticinae: Saara). Zool J Linn Soc. 2021;192(4):1123–36. doi: 10.1093/zoolinnean/zlaa113

[96] Ghane-Ameleh S, Khosravi M, Saberi-Pirooz R, Ebrahimi E, Aghbolaghi MA, Ahmadzadeh F. Mid-Pleistocene Transition as a trigger for diversification in the Irano-Anatolian region: evidence revealed by phylogeography and distribution pattern of the eastern three-lined lizard. Global Ecology and Conservation. 2021;31:e01839. doi: 10.1016/j.gecco.2021.e01839

[97] Kapli P, Botoni D, Ilgaz C, Kumlutaş Y, Avcı A, Rastegar-Pouyani N, et al. Molecular phylogeny and historical biogeography of the Anatolian lizard Apathya (Squamata, Lacertidae). Mol Phylogenet Evol. 2013;66(3):992–1001. doi: 10.1016/j.ympev.2012.12.002 23261710

[98] Carvajal-Quintero J, Villalobos F, Oberdorff T, Grenouillet G, Brosse S, Hugueny B, et al. Drainage network position and historical connectivity explain global patterns in freshwater fishes’ range size. Proc Natl Acad Sci U S A. 2019;116(27):13434–9. doi: 10.1073/pnas.1902484116 31209040 PMC6613146

[99] Patimar R, Mohammadzadeh B. On the biological characteristics of Capoeta fusca Nikolskii, 1897 in eastern Iran: biological characteristics of Capoeta fusca. J Appl Ichthyol. 2011;27(3):873–8. doi: 10.1111/j.1439-0426.2010.01572.x

[100] Emami H, et al. Structure of the mountain front flexure along the Anaran anticline in the Pusht-e Kuh Arc (NW Zagros, Iran): insights from sand box models. Geol Soc Lond Spec Publ. 2010;330(1):155–78. doi: 10.1144/SP330.9

[101] Ahmadzadeh F, Flecks M, Torki F, Bohme W. A new species of angular-toed gecko, genus Cyrtopodion (Squamata: Gekkonidae), from southern Iran. Zootaxa. 2011;2924(1). doi: 10.11646/zootaxa.2924.1.2

[102] Krause V, Ahmadzadeh F, Moazeni M, Wagner P, Wilms TM. A new species of the genus Tropiocolotes Peters, 1880 from western Iran (Squamata: Sauria: Gekkonidae). Zootaxa. 2013;3716:22–38. doi: 10.11646/zootaxa.3716.1.2 26106762

[103] Regalado L, Hernández A, Serguera M, Gómez-Hechavarría JL, Beck A. Integrative taxonomy supports the recognition of four taxa in the Notholaena trichomanoides complex (Pteridaceae) in Cuba. Biol J Linn Soc. 2023;140(3):358–75. doi: 10.1093/biolinnean/blad038

[104] Faddagh MS, Husain NA, Al-Badran AI. Usage mitochondrial 16S rRNA gene as molecular marker in taxonomy of cyprinin fish species (Cyprinidae: Teleostei). J King Abdulaziz Univ Mar Sci. 2012;23(1):39-49.

[105] Fricke R, Bilecenoglu M, Sari H. Annotated checklist of fish and lamprey species (Gnathostomata and Petromyzontomorphi) of Turkey, including a Red List of threatened and declining species. Stuttg Beitr Zur Naturkunde. 2007;706:1–172.

[106] Almaça C. Evolutionary, biogeographical, and taxonomic remarks on Mesopotamian species of Barbus ss. 1991.

[107] Kuru M. Recent systematic status of inland water fishes of Turkey. GÜ Gazi Eğitim Fakültesi Dergisi. 2004.

[108] Hipsley CA, Müller J. Beyond fossil calibrations: Realities of molecular clock practices in evolutionary biology. Front Genet. 2014;5:138. doi: 10.3389/fgene.2014.00138 24904638 PMC4033271

[109] Sauquet H. A practical guide to molecular dating. Comptes Rendus Palevol. 2013;12(6):355–67. doi: 10.1016/j.crpv.2013.07.003

[110] Khoshnamvand H, Vaissi S, Azimi M, Ahmadzadeh F. Phylogenetic climatic niche evolution and diversification of the Neurergus species (Salamandridae) in the Irano-Anatolian biodiversity hotspot. Ecol Evol. 2024;14(8):e70105. doi: 10.1002/ece3.70105 39100205 PMC11294440

[111] Rundle HD, Nosil P. Ecological speciation. Ecol Lett. 2005;8(3):336–52. doi: 10.1111/j.1461-0248.2004.00715.x

[112] Thomas CD, Cameron A, Green RE, Bakkenes M, Beaumont LJ, Collingham YC, et al. Extinction risk from climate change. Nature. 2004;427(6970):145–8. doi: 10.1038/nature02121 14712274

[113] Ahmadi M, et al. The legacy of Eastern Mediterranean mountain uplifts: rapid disparity of phylogenetic niche conservatism and divergence in mountain vipers. BMC Ecol Evol. 2021;21(1):130. doi: 10.1186/s12862-021-01863-0 34157982 PMC8220690

[114] Ahmadzadeh F, et al. Separate histories in both sides of the Mediterranean: phylogeny and niche evolution of ocellated lizards. J Biogeogr. 2016;43(6):1242–53. doi: 10.1111/jbi.12703

[115] Chase PD. Meristics. Stock Identification Methods. Elsevier. 2014. p. 171–84. doi: 10.1016/B978-0-12-397003-9.00009-6

[116] Khoshnamvand H, Malekian M, Keivani Y. Feasibility of using geometric morphometrics on larvae of Loristan newt for population identifications. J Anim Res. 2019;32(1):11–9.

[117] Zamani-Faradonbe M, Keivany Y, Khoshnamvand H. Length-weight and length-length relationships of four Garra species from Iranian basins. J Appl Ichthyol. 2018;34(6):1376–8. doi: 10.1111/jai.13809

[118] Turan C, Oral M, Öztürk B, Düzgüneş E. Morphometric and meristic variation between stocks of bluefish (Pomatomus saltatrix) in the Black, Marmara, Aegean and northeastern Mediterranean seas. Fish Res. 2006;79(1–2):139–47. doi: 10.1016/j.fishres.2006.01.015

[119] Krpo-Ćetković J, Stamenković S. Morphological differentiation of the pikeperch Stizostedion lucioperca (L.) populations from the Yugoslav part of the Danube. Ann Zool Fenn. 1996;33(3/4):711–23.

