# Supplementary material for: Climatic and geological drivers of diversity in Iranian Barbels lineage (Cypriniformes: Cyprinidae: Barbinae and Torinae): An integrative taxonomic perspective
Source: PLoS One. 2026 Jun 11;21(6):e0349868. doi: 10.1371/journal.pone.0349868 (PMC13258020; doi:10.1371/journal.pone.0349868)
Supplement: S1 Table — (PDF) [file pone.0349868.s001.pdf]

species	Accession number	Cytb	Co1
Arabibarbus grypus	AF145945	✓	
Arabibarbus grypus	KP712259	✓	
Arabibarbus grypus	AF145945	✓	
Arabibarbus grypus	KM590450		✓
Arabibarbus grypus	KM590449		✓
Arabibarbus grypus	KM590451		✓
Luciobarbus conocephalus	MZ063684	✓	
Luciobarbus conocephalus	MW649312		✓
Luciobarbus brachycephalus	MW649297		✓
Luciobarbus brachycephalus	MW649296		✓
Luciobarbus brachycephalus	KP712567	✓	
Luciobarbus esocinus	KP712264	✓	
Luciobarbus esocinus	MF599074		✓
Luciobarbus esocinus	MF599073		✓
Luciobarbus esocinus	KM590441		✓
Luciobarbus mursa	MF106172		✓
Luciobarbus mursa	MF106171		✓
Luciobarbus kersin	MF599072		✓
Luciobarbus barbulus	MF599075		✓
Luciobarbus barbulus	KM590437		✓
Luciobarbus barbulus	KM590436		✓
Luciobarbus subquintanus	AF145937	✓	
Luciobarbus xanthopterus	MF599076		✓
Luciobarbus xanthopterus	KM590446		✓
Luciobarbus xanthopterus	KM590444		✓

Mesopotamichthys sharpeyi	KF876033	✓	
Mesopotamichthys sharpeyi	KF876032	✓	
Mesopotamichthys sharpeyi	KM590447		✓
Carasobarbus luteus	FN821722	✓	
Carasobarbus luteus	FN821721	✓	
Carasobarbus luteus	FN821720	✓	
Carasobarbus luteus	MW250388		✓
Carasobarbus luteus	KM590424		✓
Carasobarbus luteus	KM590425		✓
Carasobarbus sublimus	FN821737	✓	
Carasobarbus sublimus	FN821736	✓	
Carasobarbus sublimus	KM590427		✓
Carasobarbus sublimus	KM590428		✓
Carasobarbus kosswigi	FN821741	✓	
Carasobarbus kosswigi	KU524935	✓	
Carasobarbus kosswigi	KM590423		✓
Barbus lacerta	MZ547291	✓	
Barbus lacerta	MZ547292	✓	
Barbus lacerta	MZ5472935	✓	
Barbus lacerta	MZ547287	✓	
Barbus lacerta	AF145935	✓	
Barbus lacerta	MF106136		✓
Barbus lacerta	MF106133		✓
Barbus lacerta	MF106123		✓
Barbus miliaris	MF106150		✓
Barbus miliaris	MF106146		✓
Barbus miliaris	MF106147		✓
Barbus karunensis	MF106109		✓
Barbus karunensis	MF106104		✓
Barbus karunensis	MF106099		✓
Barbus cyri	MF599081		✓
Barbus cyri	MF599084		✓
Barbus cyri	MF599086		✓
Barbus cyri	MF599088		✓

Luciobarbus capito	MW564397		✓
HK 03	PX418093	✓	
HK 04	PX418094	✓	
HK 05	PX418095	✓	
HK 12	PX418096	✓	
HK 15	PX418078	✓	
HK 18	PX418097	✓	
HK 19	PX418098	✓	
HK 21	PX418099	✓	
HK 22	PX418100	✓	
HK 25	PX418101	✓	
HK 26	PX418102	✓	
HK 27	PX418103	✓	
HK 30	PX418079	✓	
HK 33	PX418080	✓	
HK 35		✓	
HK 37		✓	
HK 38	PX418081	✓	
HK 40	PX418082	✓	
HK 42	PX418083	✓	
HK 45	PX418084	✓	
HK 46	PX418085	✓	
HK 47	PX418086	✓	
HK 50	PX418087	✓	
HK 51	PX418088	✓	
HK 53	PX418089	✓	
HK 54	PX418090	✓	
HK 55	PX418091	✓	
HK 61	PX418092	✓	
Garra rufa	MN340167	✓	
Garra rufa	JF416297		✓
Capoeta capoeta	MF664749	✓	
Capoeta capoeta	MF664711		✓
