# Supplementary material for: Climatic and geological drivers of diversity in Iranian Barbels lineage (Cypriniformes: Cyprinidae: Barbinae and Torinae): An integrative taxonomic perspective
Source: PLoS One. 2026 Jun 11;21(6):e0349868. doi: 10.1371/journal.pone.0349868 (PMC13258020; doi:10.1371/journal.pone.0349868)
Supplement: S2 Table — (PDF) [file pone.0349868.s002.pdf]

	***A.grypus* (n=7)**	***M.sharpie* (n=3)**	***C.kosswigi* (n=5)**	***C.luteus* (n=5)**	***C.sublimus*** (n=4)	***B.karunens*** (n=3)	***B.lacerta*** (n=10)	***B.miliaris*** (n=4)	***B.cyri***(n=6)	***L.barbulus*** (n=10)	***L.capito*** (n=4)	***L.brachycephalus*** (n=3)	***L.conocephalus*** (n=3)	***L.esocinus*** (n=7)	***L.xanthopterus*** (n=4)	***L.kersin*** (n=3)	***L.mursa*** (n=6)	***L.subquincunciatus*** (n=3)
Dorsal fin unbranched rays **(Dfur)**	3-4±0.46	4±0.00	4±0.00	4±0.00	3-4±0.5	4±0.00	3-5±0.64	4±0.00	3-5±0.89	3-4±0.51	4-5±0.5	3-4±0.7	4±0.00	4±0.00	4±0.00	4±0.00	4-5±0.5	4±0.00
Dorsal fin branched rays **(Dfbr)**	7-8±0.46	8±0.00	9-10±0.44	9-10±0.48	11±0.00	8±0.00	7-8±0.46	8±0.00	8±0.00	8-9±0.54	8±0.00	7±0.00	8±0.00	8±0.00	8±0.00	8±0.00	7-8±0.5	8±0.00
Anal fin unbranched rays **(Afur)**	3-4±0.46	3±0.00	3±0.00	3±0.00	3±0.00	3±0.00	8±0.00	3-4±0.57	3-4±0.44	3±0.00	3-4±0.5	3±0.00	3±0.00	3±0.00	3±0.00	3±0.00	3±0.00	3±0.00
Anal fin branched rays**(Afbr)**	5-6±0.35	5±0.00	6±0.00	6±0.00	6±0.00	5±0.00	3±0.00	5±0.00	5±0.00	5±0.00	5±0.00	5±0.00	5±0.00	5±0.00	5±0.00	5±0.00	5±0.00	5±0.00
Pectoral fin branched rays**(Pfbr)**	15-16±0.35	16±0.00	15-16±0.54	16-16±0.48	16-17-4±0.5	15±0.00	15-17±0.64	15-16±0.57	15-16±0.44	16-17±0.4	16-17±0.00	15-16±0.7	17±0.00	17±0.00	17-18±0.5	17±0.00	15-17±1	16±0.00
Lateral line scales(Lls)	39-41±0.7	32±0.00	33±0.00	33-34±0.53	27-28±0.5	67±0.00	65-66±0.37	79±0.00	66-67±0.44	58-59±0.4	70±0.00	75±0.00	65±0.46	69-70±0.37	69±0.00	54±0.00	91-93±1	80±0.00
