# Supplementary material for: Climatic and geological drivers of diversity in Iranian Barbels lineage (Cypriniformes: Cyprinidae: Barbinae and Torinae): An integrative taxonomic perspective
Source: PLoS One. 2026 Jun 11;21(6):e0349868. doi: 10.1371/journal.pone.0349868 (PMC13258020; doi:10.1371/journal.pone.0349868)
Supplement: S3 Table — (PDF) [file pone.0349868.s003.pdf]

**Species**	**Lat**	**Long**	**Location**
A.grypus	33.14	44.27	Tigris
A.grypus	33.17	44.12	Tigris
A.grypus	33.14	44.27	Tigris
A.grypus	33.14	44.27	Tigris
A.grypus	33.293	48.005	Tigris
A.grypus	33.294	47.5821	Tigris
A.grypus	32.0805	48.1241	Tigris
A.grypus	32.2427	48.0854	Tigris
A.grypus	32.23	42.14	Tigris
A.grypus	28.13	55.22	Hormoz
A.grypus	29.17	51.96	Persis
A.grypus	28.99	51.85	Persis
Carasobarbus kosswigi	33.2423	47.5742	Tigris
Carasobarbus kosswigi	33.2423	47.5742	Tigris
Carasobarbus kosswigi	33.2423	47.5742	Tigris
Carasobarbus kosswigi	33.2448	47.5756	Tigris
Carasobarbus kosswigi	33.24	47.5756	Tigris
Carasobarbus kosswigi	33.3918	47.0221	Tigris
Carasobarbus luteus	33.0354	48.381	Tigris
Carasobarbus luteus	33.0323	48.3833	Tigris
Carasobarbus luteus	32.0439	48.1259	Tigris
Carasobarbus luteus	32.0439	48.1259	Tigris
Carasobarbus luteus	31.572	48.1359	Tigris
Carasobarbus luteus	33.1854	47.5224	Tigris
Carasobarbus luteus	28.4131	51.4848	Persis
Carasobarbus luteus	2956	50.89	Persis
Carasobarbus luteus	28.64	55.3	Hormoz
Carasobarbus Sublinus	32.1033	49.0702	Tigris
Carasobarbus Sublinus	33.1641	47.5994	Tigris
Carasobarbus Sublinus	34.5546	46.5626	Tigris
Carasobarbus Sublinus	32.74	48.51	Tigris
Carasobarbus Sublinus	33.69	47.77	Tigris
Carasobarbus Sublinus	31.58	48.31	Tigris
Mesopotamichthys sharpeyi	33.1	47.53	Zohreh
Mesopotamichthys sharpeyi	30.66	48.34	Tigris
Mesopotamichthys sharpeyi	31.43	48.84	Tigris
Mesopotamichthys sharpeyi	30.90	48.81	Tigris
Mesopotamichthys sharpeyi	31.64	48.78	Tigris
Luciobarbus barbulus	33.451	48.2628	Tigris
Luciobarbus barbulus	33.451	48.2628	Tigris
Luciobarbus barbulus	31.5486	51.2068	Tigris
Luciobarbus barbulus	34.5167	45.5905	Tigris

Luciobarbus barbulus	28.9713	54.3905	Persis
Luciobarbus barbulus	28.16	53.33	Persis
Luciobarbus barbulus	28.58	52.56	Persis
Luciobarbus barbulus	33.54	46.88	Tigris
Luciobarbus barbulus	33.78	47.44	Tigris
Luciobarbus barbulus	33.37	47.92	Tigris
Luciobarbus barbulus	33.90	46.76	Tigris
Luciobarbus barbulus	33.22	47.38	Tigris
Luciobarbus barbulus	35.15	46.16	Tigris
Luciobarbus barbulus	32.12	49.35	Zohreh
Luciobarbus capito	39.13	46.93	Caspian
Luciobarbus capito	38.39	48.22	Caspian
Luciobarbus capito	37.54	48.34	Caspian
Luciobarbus capito	37.20	49.14	Caspian
Luciobarbus capito	36.99	49.58	Caspian
Luciobarbus capito	36.16	52.95	Caspian
Luciobarbus esocinus	33.451	48.2628	Tigris
Luciobarbus esocinus	32.4419	48.0346	Tigris
Luciobarbus esocinus	32.5825	47.4816	Tigris
Luciobarbus esocinus	32.5825	47.4816	Tigris
Luciobarbus esocinus	31.52	47.93	Tigris
Luciobarbus esocinus	33.06	46.67	Tigris
Luciobarbus esocinus	33.09	47.57	Tigris
Luciobarbus xanthopterus	32.5825	47.4816	Tigris
Luciobarbus xanthopterus	31.52	47.93	Tigris
Luciobarbus xanthopterus	33.06	46.67	Tigris
Luciobarbus xanthopterus	33.09	47.57	Tigris
Luciobarbus xanthopterus	31.63	48.76	Tigris
Luciobarbus mursa	39.23	46.93	Caspian
Luciobarbus mursa	38.37	48.22	Caspian
Luciobarbus mursa	37.56	48.36	Caspian
Luciobarbus mursa	37.28	49.14	Caspian
Luciobarbus mursa	36.93	49.54	Caspian
Luciobarbus mursa	36.14	52.97	Caspian
Luciobarbus mursa	36.13	52.41	Caspian
Luciobarbus mursa	36.63	54.12	Caspian
Barbus lacerta	35.1177	46.2568	Tigris
Barbus lacerta	32.23	42.14	Tigris
Barbus lacerta	36.4767	45.3317	Tigris
Barbus lacerta	35.1603	46.3394	Tigris
Barbus lacerta	33.2448	33.2448	Tigris
Barbus lacerta	33.1383	49.6789	Tigris
Barbus lacerta	33.4016	47.0323	Tigris
Barbus lacerta	34.9008	46.2097	Tigris
Barbus lacerta	34.5544	47.4133	Tigris
Barbus lacerta	35.0421	46.3545	Tigris

Barbus lacerta	33.7844	48.2069	Tigris
Barbus lacerta	34.3838	47.7663	Tigris
Barbus lacerta	34.5945	48.7389	Tigris
Barbus lacerta	33.2448	33.2448	Tigris
Barbus lacerta	32.0296	50.6294	Kor
Barbus lacerta	33.51	49.93	Namak
Barbus lacerta	33.451	48.2628	Tigris
Barbus lacerta	33.2046	47.5528	Tigris
barbus cyri	37.4167	54.1490	Urmia
barbus cyri	36.9949	45.0716	Urmia
barbus cyri	38.24	44.99	Urmia
barbus cyri	37.3012	45.1203	Urmia
barbus cyri	35.7429	46.4446	Urmia
barbus cyri	38.00	47.16	Urmia
barbus cyri	38.0025	47.7611	Urmia
barbus cyri	36.53	46.49	Urmia
barbus cyri	36.8148	50.8740	Caspian
barbus cyri	36.42	48.36	Caspian
barbus cyri	36.2814	53.2385	Caspian
barbus cyri	37.507	47.907	Caspian
barbus cyri	37.25	49.39	Caspian
barbus miliaris	34.8874	50.0376	Namak
barbus miliaris	35.58	50.62	Namak
barbus miliaris	34.3531	50.5480	Namak
barbus miliaris	34.84	51.75	Namak
barbus miliaris	35.3019	52.4160	Kavir
barbus karunensis	31.1837	51.2671	Kor
barbus karunensis	31.1840	51.4498	Esfahan
barbus karunensis	31.2615	51.2858	Tigris
barbus karunensis	31.2615	51.285	Tigris
barbus karunensis	32.0114	50.6114	Esfahan
Luciobarbus brachycephalus	37.26	49.43	Caspian
Luciobarbus brachycephalus	37.40	48.84	Caspian
Luciobarbus brachycephalus	38.35	48.34	Caspian
Luciobarbus conocephalus	35.94	71.17	Harir
Luciobarbus conocephalus	35.62	61.26	Harir
Luciobarbus conocephalus	36.26	61.21	Harir
Luciobarbus kersin	31.45	47.77	Tigris
Luciobarbus kersin	31.07	47.67	Tigris

Luciobarbus subquincunciatus	32.43	48.46	Tigris
Luciobarbus subquincunciatus	33.24	47.77	Tigris
Luciobarbus subquincunciatus	32.62	47.44	Tigris
Luciobarbus subquincunciatus	33.33	47.20	Tigris
