# Supplementary material for: Climatic and geological drivers of diversity in Iranian Barbels lineage (Cypriniformes: Cyprinidae: Barbinae and Torinae): An integrative taxonomic perspective
Source: PLoS One. 2026 Jun 11;21(6):e0349868. doi: 10.1371/journal.pone.0349868 (PMC13258020; doi:10.1371/journal.pone.0349868)
Supplement: S4 Table — (PDF) [file pone.0349868.s004.pdf]

Node	Original calibration (30 Mya, lognormal)	Alternative 1: No secondary calibration (loose upper bound 90 Mya)	Alternative 2: Alternative secondary calibration (28 Mya, Wang et al. 2013)	Alternative 3: Uniform prior (25–35 Mya)
**Basal divergence of Barbus group (Node A)**	34.3 (26.8–40.7)	36.1 (27.5–44.2)	32.8 (25.1–39.5)	33.9 (26.2–41.0)
**Divergence between Group I (*Luciobarbus* + *Barbus* s.str.) and Group II (*Carasobarbus* + *Arabibarbus* + *Mesopotamichthys*)**	34.3 (26.8–40.7)	36.1 (27.5–44.2)	32.8 (25.1–39.5)	33.9 (26.2–41.0)
**Divergence between *Luciobarbus* and *Barbus* s.str. (Node B)**	21.1 (18.6–26.1)	22.5 (19.2–28.3)	20.3 (17.4–25.0)	20.8 (18.0–25.8)
**Divergence within *Luciobarbus* clade (Node C)**	12.3 (8.1–13.9)	13.1 (8.9–15.2)	11.8 (7.6–13.5)	12.0 (7.9–13.7)
**Divergence within *Barbus* s.str. clade (Node D)**	11.4 (7.5–14.7)	12.2 (8.1–15.9)	10.9 (7.0–14.1)	11.1 (7.3–14.5)
Divergence between L. esocinus and L. xanthopterus	0.8 (0.4–1.2)	0.9 (0.5–1.4)	0.7 (0.3–1.1)	0.8 (0.4–1.3)
**Divergence between *B. lacerta* and *B. cyri***	1.5 (0.9–2.2)	1.7 (1.0–2.5)	1.4 (0.8–2.1)	1.5 (0.9–2.3)
