# Supplementary material for: Climatic and geological drivers of diversity in Iranian Barbels lineage (Cypriniformes: Cyprinidae: Barbinae and Torinae): An integrative taxonomic perspective
Source: PLoS One. 2026 Jun 11;21(6):e0349868. doi: 10.1371/journal.pone.0349868 (PMC13258020; doi:10.1371/journal.pone.0349868)
Supplement: S5 Table — (PDF) [file pone.0349868.s005.pdf]

PC	Eigenvalue	Variance (%)	Cumulative (%)
1	3.1247	44.64	44.64
2	1.8521	26.46	71.10
3	0.9876	14.11	85.21
4	0.5432	7.76	92.97
5	0.28	4.00	96.97
6	0.15	2.14	99.11
7	0.06	0.89	100.00
