# Supplementary material for: Climatic and geological drivers of diversity in Iranian Barbels lineage (Cypriniformes: Cyprinidae: Barbinae and Torinae): An integrative taxonomic perspective
Source: PLoS One. 2026 Jun 11;21(6):e0349868. doi: 10.1371/journal.pone.0349868 (PMC13258020; doi:10.1371/journal.pone.0349868)
Supplement: S6 Table — Values shown are means ± standard deviation (SD) across 10 bootstrap replicates. Only species with ≥5 unique occurrence localities were modeled. BIO1 = Annual Mean Temperature, BIO7 = Temperature Annual Range, BIO14 = Precipitation of Driest Month. AUC = Area Under the Curve; TSS = True Skill Statistic. Dashes (—) indicate that the species was excluded due to insufficient sample size. (PDF) [file pone.0349868.s006.pdf]

Species	N	AUC (mean ± SD)	TSS (mean ± SD)	BIO1 (%)	BIO7 (%)	BIO14 (%)
**Torinae**						
Carasobarbus luteus	8	0.89 ± 0.04	0.71 ± 0.06	24.3	18.7	**57.0**
Carasobarbus kosswigi	5	0.85 ± 0.07	0.63 ± 0.11	31.2	22.4	**46.4**
Carasobarbus sublimus	7	0.91 ± 0.03	0.74 ± 0.05	19.8	15.3	**64.9**
Arabibarbus grypus	12	0.93 ± 0.02	0.79 ± 0.04	22.1	19.6	**58.3**
Mesopotamichthys sharpeyi	6	0.86 ± 0.06	0.65 ± 0.09	28.4	20.1	**51.5**
**Barbinae**						
Luciobarbus mursa	9	0.94 ± 0.03	0.81 ± 0.05	18.2	**45.6**	36.2
Luciobarbus brachycephalus	7	0.88 ± 0.05	0.69 ± 0.08	25.7	**41.3**	33.0
Luciobarbus capito	15	0.91 ± 0.03	0.76 ± 0.04	21.5	29.8	**48.7**
Luciobarbus conocephalus	6	0.84 ± 0.08	0.61 ± 0.12	29.1	27.4	**43.5**
Luciobarbus barbulus	10	0.92 ± 0.02	0.78 ± 0.03	20.3	24.7	**55.0**
Luciobarbus kersin	5	0.83 ± 0.09	0.59 ± 0.14	26.8	30.2	**43.0**
Luciobarbus esocinus	14	0.90 ± 0.04	0.75 ± 0.06	19.4	28.5	**52.1**
Luciobarbus xanthopterus	11	0.89 ± 0.04	0.73 ± 0.07	22.7	26.9	**50.4**
Barbus lacerta	18	0.92 ± 0.03	0.77 ± 0.05	23.4	25.1	**51.5**
Barbus cyri	8	0.87 ± 0.05	0.68 ± 0.08	27.9	24.3	**47.8**
Barbus karunensis	5	0.82 ± 0.10	0.56 ± 0.15	30.5	26.7	**42.8**
Barbus miliaris	6	0.88 ± 0.06	0.67 ± 0.10	24.6	22.9	**52.5**
**Excluded (n < 5)**						
Luciobarbus subquincunciatus	3	–	–	–	–	–
