# Supplementary material for: Climatic and geological drivers of diversity in Iranian Barbels lineage (Cypriniformes: Cyprinidae: Barbinae and Torinae): An integrative taxonomic perspective
Source: PLoS One. 2026 Jun 11;21(6):e0349868. doi: 10.1371/journal.pone.0349868 (PMC13258020; doi:10.1371/journal.pone.0349868)
Supplement: S7 Table — (PDF) [file pone.0349868.s007.pdf]

**Species group comparison**	**Mean Schoener's D (± SD)**	**Mean Hellinger's I (± SD)**	**Range (D)**
Torinae clade (*Carasobarbus* + *Arabibarbus* + *Mesopotamichthys*)	0.68 ± 0.06	0.72 ± 0.05	0.61–0.78
Luciobarbus (southern spp.: *L. xanthopterus, L. esocinus, L. barbulus, L. kersin*)	0.83 ± 0.04	0.87 ± 0.03	0.76–0.89
Luciobarbus (Northern spp.: *L. capito, L. mursa*) vs. Southern Luciobarbus	0.42 ± 0.31	0.46 ± 0.29	0.10–0.84
Barbus (str) clade (*B. lacerta, B. cyri*)	0.76	0.83	_
Cross-clade (torinae vs. *Luciobarbus* vs. *Barbus* s.str.)	0.19 ± 0.07	0.22 ± 0.08	0.09–0.35
