# Supplementary figures and images for: Climatic and geological drivers of diversity in Iranian Barbels lineage (Cypriniformes: Cyprinidae: Barbinae and Torinae): An integrative taxonomic perspective
Source: PLoS One. 2026 Jun 11;21(6):e0349868. doi: 10.1371/journal.pone.0349868 (PMC13258020; doi:10.1371/journal.pone.0349868)

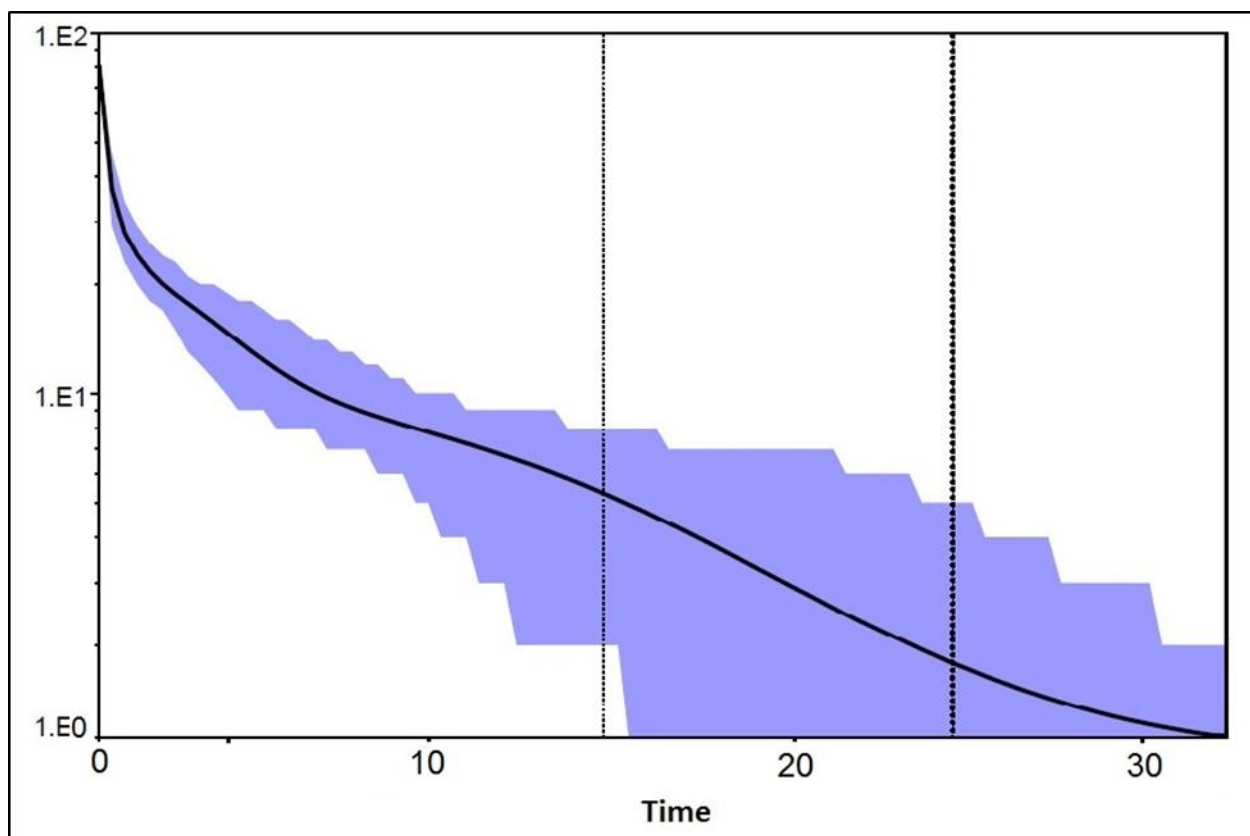

Supplement: S8 Fig — The raw axis is time in millions of years. (PDF) [file pone.0349868.s008.pdf]
